# Effects of Pump Photon Energy on Generation and Ultrafast Relaxation of Excitons and Charge Carriers in CdSe Nanoplatelets

**DOI:** 10.1021/acs.jpcc.2c07292

**Published:** 2023-01-19

**Authors:** Michele Failla, Fransisco García Flórez, Bastiaan B. V. Salzmann, Daniel Vanmaekelbergh, Henk T. C. Stoof, Laurens D. A. Siebbeles

**Affiliations:** † Chemical Engineering Department, Delft University of Technology, Van der Maasweg 9, 2629 HZDelft, The Netherlands; ‡ Institute for Theoretical Physics and Center for Extreme Matter and Emergent Phenomena, Utrecht University, Princetonplein 5, 3584 CCUtrecht, The Netherlands; § Condensed Matter and Interfaces, Debye Institute, Utrecht University, Princetonplein 1, 3584 CCUtrecht, The Netherlands

## Abstract

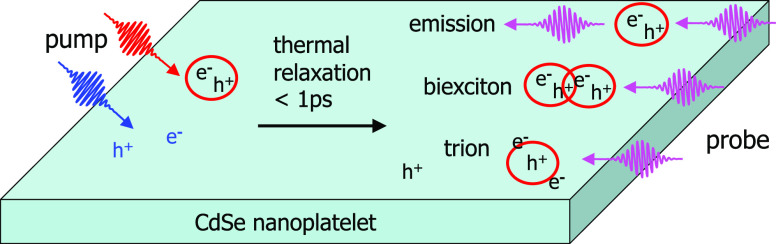

We studied the initial nature and relaxation of photoexcited electronic states in CdSe nanoplatelets (NPLs). Ultrafast transient optical absorption (TA) measurements were combined with the theoretical analysis of the formation and decay of excitons, biexcitons, free charge carriers, and trions. In the latter, photons and excitons were treated as bosons and free charge carriers as fermions. The initial quantum yields of heavy-hole (HH) excitons, light-hole (LH) excitons, and charge carriers vary strongly with photon energy, while thermal relaxation occurs always within 1 ps. After that, the population of LH excitons is negligible due to relaxation to HH excitons or decay into free electrons and holes. Up to the highest average number of about four absorbed photons per NPL in our experiments, we found no signatures of the presence of biexcitons or larger complexes. Biexcitons were only observed due to the interaction of a probe-generated exciton with an exciton produced previously by the pump pulse. For higher pump photon energies, the initial presence of more free charge carriers leads to formation of trions by probe photons. On increasing the number of absorbed pump photons in an NPL, the yield of excitons becomes higher as compared to free charge carriers, since electron–hole recombination becomes more likely. In addition to a TA absorption feature at energy below the HH exciton peak, we also observed a TA signal at the high-energy side of this peak, which we attribute to formation of LH-HH biexcitons or trions consisting of a charge and LH exciton.

## Introduction

Metal chalcogenide nanoplatelets (NPLs) with an atomically precise thickness of a few nanometers and finite lateral dimensions of the order of tens of nanometers can be synthesized by colloidal chemistry approaches.^1^ The liquid phase colloidal synthesis allows one to control the composition, size, and shape of NPLs and has prospect for cheap production on a large scale. CdSe and several other metal chalcogenide NPLs exhibit robust excitons with narrow excitonic optical absorption and photoluminescence (PL) peaks, large optical oscillator strength, and high PL quantum yield.^2^ These properties offer promising prospect for optoelectronic and photocatalytic applications.^2^

Photogeneration, properties, and decay of excitons and free charge carriers in CdSe NPLs have been studied using a variety of time-resolved pump-probe laser spectroscopy techniques, as recently reviewed by Hu et al.^1^ Transient optical absorption (TA) spectroscopy has been used to study charge carrier cooling and exciton formation,^3−5^ decay by Auger recombination,^6,7^ charge transfer,^8−11^ optical gain,^4,5,12^ and the relation between exciton localization and radiative lifetime.^13^ Information about the quantum yields of thermally equilibrated excitons and free charge carriers has been obtained from the theoretical analysis of measured TA and optical-pump terahertz-probe spectroscopy data after photoexcitation of CdSe NPLs.^4,14^ According to the latter studies, even after photoexcitation well above the HH exciton energy, thermal relaxation leads mainly to formation of excitons rather than free charge carriers. The aim of the current work is to determine the effects of pump photon energy on the initial nature of excitons and charge carriers in CdSe NPLs, as well as their ultrafast thermal relaxation.

In Results and Discussion, we first discuss the origin of the features in the steady-state optical absorbance spectrum. Next, we address the effects of pump photon energy and the average number of absorbed pump photons in an NPL on the TA spectra on a timescale from hundreds of femtoseconds up to nanoseconds after the pump pulse. We then introduce a theoretical model to describe the photogeneration and thermal relaxation of excitons, biexcitons, charge carriers, and trions. Our model gives an excellent description of the experimental results and treats photons and excitons explicitly as bosons, while free charge carriers are described as fermions. Details of the model are presented in the Supporting Information. We use our model to analyze the TA spectra and to obtain information about contributions from excitons, biexcitons, free charge carriers, and trions at an ultrashort time of a few hundred femtoseconds. Finally, the theoretical analysis is extended to a nanosecond timescale.

## Experimental Section

We synthesized CdSe NPLs with a thickness of 4.5 monolayers and lateral dimensions of (25 ± 3) × (8 ± 1) nm^2^, as described previously. The NPLs were dispersed in hexane in an airtight Hellma QS cuvette inside a nitrogen-purged glovebox to avoid contact with air.^15^ The Fermi level of such samples is near the middle of the NPL band gap, which implies that unintentional doping and the presence of background charge carriers are negligible.^16^ The steady-state optical absorbance spectrum, *A*_0_(*ℏ*ω), as a function of photon energy *ℏ*ω was obtained with a double-beam PerkinElmer Lambda 1050 UV/Vis spectrometer.

Transient optical absorption (TA) measurements were performed on solutions of CdSe NPLs dispersed in hexane inside an air-tight quartz cuvette. A Yb-KGW oscillator (Light Conversion, Pharos SP) was used to produce 180 fs photon pulses with a wavelength of 1028 nm and at a frequency of 5 kHz. Pump pulses at other wavelengths were obtained by nonlinear frequency mixing of the fundamental beam through an Optical Parametric Amplifier equipped with a second harmonic module (Light Conversion, Orpheus). A small fraction of the 1028 fundamental beam was directed into a sapphire crystal to produce probe photons in the range of 500–1600 nm. The pump beam was transmitted through a mechanical chopper operating at 2.5 kHz, allowing one in every two pump pulses to be transmitted. Pump and probe beams overlap at the sample position with a small angle of about 8°, and they arrive at a relative time delay controlled by an automated delay stage. After transmission through the sample, the pump pulses are dumped, while the probe pulses are collected at a detector (Ultrafast Systems, Helios). The probe spectra are corrected for dispersion by fitting a polynomial to the solvent response. During TA measurements, the NPL dispersion was stirred to avoid effects of NPL degradation. The TA measurements provide the difference of the absorbance of the sample at probe photon energies *E*_p_ at time *t* after the pump pulse, *A*_on_(*E*_p_, *t*), and that without the pump pulse, *A*_off_(*E*_p_), according to Δ*A*(*E*_p_, *t*) = *A*_on_(*E*_p_, *t*) – *A*_off_(*E*_p_) (see also Section S2). The optical absorbance spectrum after the pump pulse is obtained from *A*_on_(*E*_p_, *t*) = *A*_0_(*E*_p_) + Δ*A*(*E*_p_, *t*), where we used *A*_off_(*E*_p_) = *A*_0_(*E*_p_).

## Results and Discussion

### Steady-State Absorbance and Short-Time Dynamics of the Transient Absorption Spectra

**Figure 1:**
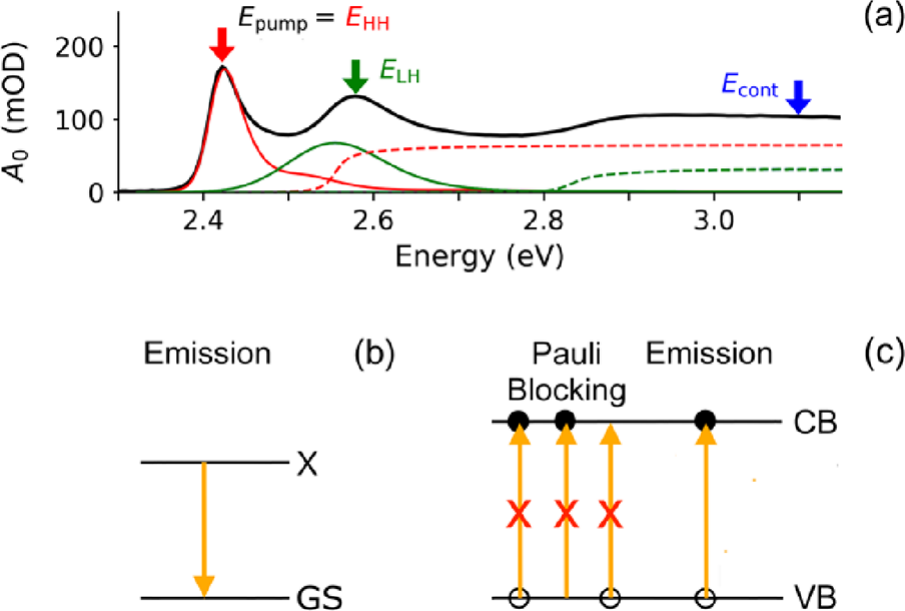
(a) Optical absorbance spectrum of the CdSe NPL dispersion. In the TA experiments, the NPLs were excited at pump photon energies, *E*_pump_, corresponding to the maxima of the peaks due to HH and LH excitons and in the continuum, as indicated by the colored arrows. The contributions of optical transitions to HH excitons (red curve) and LH excitons (green curve) in different COM states are indicated, as well as those of transitions to the corresponding continua (dashed curves). (b) Photon emission from an exciton (X) to the ground state (GS). (c) Pauli blocking and emission involving holes in the valence band (VB) and electrons in the conduction band (CB).

Figure 1a shows the steady-state optical absorbance spectrum, *A*_0_, of the NPL dispersion as a function of photon energy. The first and second peaks are due to optical excitation to heavy-hole (HH) and light-hole (LH) excitons with maxima at *E*_HH_ = 2.42 eV and *E*_LH_ = 2.58 eV, respectively. The red and green solid lines depict the contributions of exciton states with discrete energies due to the laterally confined motion of their center of mass (COM), as we obtained from theoretical analysis before.^15^ At higher energies, photoexcitation from HH or LH valence band states to conduction band states occurs.^15^ The contributions of the HH and LH continua to the optical absorbance are shown as red and green dashed curves, respectively. Note that the LH exciton peak has an energy above the onset of the HH continuum. Hence, an LH exciton can dissociate into a hole in the HH valence band and an electron in the conduction band.

**Figure 2:**
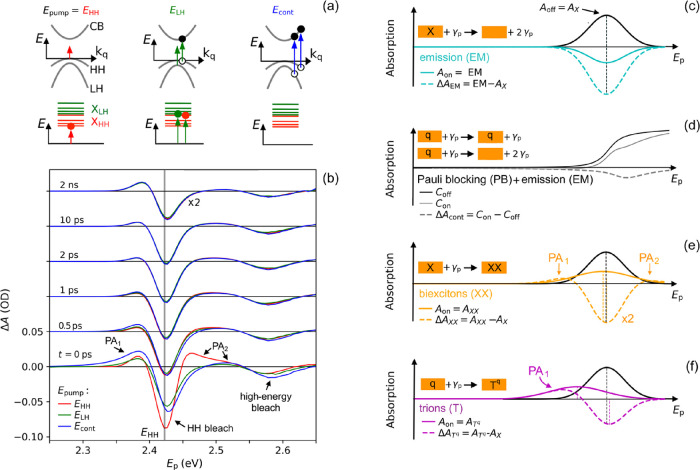
(a) Optical transitions in the valence/conduction band picture (top panels) and in the exciton picture (bottom) for pump photon energies *E*_pump_ equal to *E*_HH_ = 2.42 eV (left column), *E*_LH_ = 2.58 eV (middle column), and *E*_cont_ = 3.10 eV (right column). (b) Experimental TA spectra for *E*_pump_ energies *E*_HH_ (red curves), *E*_LH_ (green curves), and *E*_cont_ (blue curves), obtained at different delay times *t* with *N*_av_ ≃ 3. The vertical gray line indicates the energy *E*_HH_ of the HH exciton peak in Figure 1a. (c) Steady-state absorbance due to exciton formation *A*_off_ = *A*_X_ (black curve). Excitons (X) lead to photon emission (EM) induced by the probe pulse (solid cyan curve). For clarity, the picture shows an extreme case in which emission dominates over absorption after the pump pulse so that *A*_on_ is negative (cyan curve). (d) Electrons and holes (*q*) cause reduced absorption of probe photons (γ_p_) by Pauli blocking (PB) or photon emission (EM) via electron–hole recombination. The bleach (dashed gray curve) arises, since the net absorbance without pump pulse (*C*_off_, black curve) is larger than after the pump pulse when PB and EM occur (*C*_on_, gray curve). (e) Transitions to broadened HH-HH or LH-HH biexciton states (*A*_XX_, solid yellow curve) at lower and higher energies compared to *A*_X_, respectively, result in a bleach and two photoinduced absorbance features in Δ*A*_XX_ (dashed yellow curve). For clarity, Δ*A*_XX_ was scaled by a factor of 2. (f) Transition to a trion state (solid magenta curve) leads to the feature *A*_T^q^_ with the maximum at lower energy than the maximum due to the exciton transition *A*_X_ (black curve), which is qualitatively similar to that of a biexciton.

To study the initial nature and subsequent relaxation of photoexcited electronic states, we performed TA measurements (see Experimental Section) using pump photons with different energies, *E*_pump_, as indicated by the arrows in Figure 1a, where *E*_HH_ = 2.42 eV, *E*_LH_ = 2.58 eV, and *E*_cont_ = 3.10 eV. Before discussing the measured TA spectra (Figure 2b), we first describe the excitation processes that can occur at different pump photon energies (see Figure 2a).^17^ The top panels in Figure 2a correspond to a valence/conduction band picture for free charge carriers with wave vector **k**_q_ in the plane of an NPL. The bottom panels depict transitions to HH and LH excitons with discrete lateral COM motion energies, which can be described by the particle-in-a-box model.^15^ For pump photon energy *E*_HH_, predominantly, HH excitons are generated initially, since the absorbance due to excitation to HH excitons (red curve in Figure 1a) does not show significant overlap with the other optical transitions obtained from theoretical analysis.^15^ For photon energy *E*_LH_, formation of LH excitons will be dominant (see the green curve in Figure 1a). Note that the overlap of the green curve in Figure 1a with the red curves implies that also HH excitons with higher COM energy and pairs of a hole in the HH valence band and an electron in the conduction band will occur. Photons with energy *E*_cont_ produce pairs of holes in the HH and LH valence bands and electrons in the conduction band, as inferred from the overlapping dashed curves in Figure 1a and depicted in the right panel of Figure 2a.

The measured TA spectra as a function of probe photon energy *E*_p_ at different times *t* after the pump pulse, Δ*A*(*E*_p_, *t*) = *A*_on_(*E*_p_, *t*) – *A*_off_(*E*_p_), are shown in Figure 2b for pump photon energies, *E*_pump_, as indicated. The spectra *A*_on_(*E*_p_, *t*) and *A*_off_(*E*_p_) are the absorbance with and without pump pulse, respectively (see Experimental Section). Zero delay time is defined as *t* = 0 = *t*_PA_1__^max^ at which the low-energy TA feature, denoted as PA_1_, has reached maximum amplitude (see Figure S1). As can be seen in Figure S1, for all *E*_pump_, the time *t*_PA_1__^max^ is a few hundred femtoseconds after the start of the rise of the PA_1_ feature, which corresponds to the duration of the laser pulses (see Experimental Section). All data in Figure 2b (and in Figures 4 and 5) were obtained with similar values of the average number of absorbed pump photons per NPL equal to *N*_av_ ≃ 3 for each pump photon energy (see Figure S2 in Section S1). The shapes of the TA spectra initially vary with the pump photon energy, since the latter determines the photogeneration quantum yield of HH excitons, LH excitons, and charge carriers, as discussed above and illustrated in Figure 2a. After 1 ps, the spectra have the same shape for all pump photon energies, from which we infer that the initially created excitons and charge carriers have thermally relaxed to the same energy distribution, in agreement with previous results.^4,10,18^

During the entire time range from *t* = 0 to 2 ns, the TA spectrum exhibits a large bleach (Δ*A* < 0) with maximum amplitude slightly above the energy of the HH exciton peak in the steady-state absorbance spectrum, *E*_HH_, which is indicated by the vertical gray line in Figure 2b. This bleach (Δ*A*_EM_ = EM – *A*_X_ < 0) is due to photon emission (EM) from HH excitons, as depicted in Figures 1b and 2c and reported previously for CdSe NPLs.^4,5,8,10,11,13,19^ The effect of EM from excitons on the TA spectra is further discussed theoretically below and in Section S3. For a pump photon with energy *E*_HH_ = 2.42 eV, the HH bleach at *t* = 0 is more pronounced than for higher pump photon energies (see Figure 2b). This can be understood, since for photoexcitation at *E*_HH_, initially, predominantly, HH excitons are produced in contrast to the case for higher pump photon energies (see Figures 1a and 2a). The TA spectra also exhibit a high-energy bleach feature around probe photon energy *E*_LH_=2.58 eV, which persists during the entire 2 ns time range in Figure 2b. After thermal relaxation during the first picosecond, the population of LH excitons will be small, since most have relaxed to HH excitons or have decayed into electrons and holes in the HH continuum with a large density of states. The latter is corroborated by the theoretical analysis discussed below. Hence, we can attribute the high-energy bleach (Δ*A* = *C*_on_ – *C*_off_ < 0 in Figure 2d) to resulting mainly from Pauli blocking via state filling by holes in the HH valence band and electrons in the conduction band or by photon emission via radiative recombination of electrons and holes (see Figure 1c). Ascribing the high-energy bleach mainly to charge carriers agrees with the observation that its amplitude is the largest for pump photons with energy *E*_cont_ = 3.10 eV at which, initially, only charges are generated (see the TA spectra at *t* = 0 in Figure 2b).

The TA spectra in Figure 2b exhibit photoinduced absorbance features below and above the energy of the HH peak, denoted as PA_1_ and PA_2_, respectively. These features are attributed to the probe pulse producing biexcitons or trions, with excitons or charge carriers produced previously by the pump pulse. Figure 2e sketches the case of biexciton formation. The biexciton binding energy causes the absorbance profile, *A*_XX_, due to biexciton formation by the probe to shift to lower energy with respect to that for excitons, *A*_X_.^5,20^ There is also broadening as a result of the distribution of exciton–exciton and exciton–charge interactions.^20^ In addition, a tail to lower probe photon energies occurs due to the smaller COM energy dispersion of biexcitons as compared to excitons.^5^ This is due to the fact that for a larger COM momentum of an exciton, the transition to a biexciton occurs at lower probe photon energy, since the larger biexciton mass causes the COM motion energy dispersion to be weaker than for excitons. Note that it is also possible that the probe pulse produces an LH exciton that binds to an already present HH exciton, leading to formation of an LH-HH biexciton. The binding energy between the HH and LH excitons then causes an absorbance at energy above *E*_HH_ (and below *E*_LH_) and may contribute to the PA_2_ feature, as indicated in Figure 2e.

Formation of trions (see Figure 2f) by the probe pulse can also contribute to the PA_1_ and PA_2_ features, as follows. A probe photon may produce an HH or LH exciton near a charge carrier produced by the pump pulse and form a trion (T^q^) with energy below that of the exciton.^21,22^ Interestingly, Figure 2b shows that the PA_1_ feature at *t* < 1 ps is the largest for the highest pump photon energy of *E*_cont_ = 3.10 eV at which, initially, only charges are produced (see Figure 2a). Apparently, at 3.10 eV, the contribution of trion formation to the PA_1_ absorption feature compensates for the bleach due to HH excitons to a larger extent than for the lower pump photon energies *E*_HH_ and *E*_LH_.

**Figure 3:**
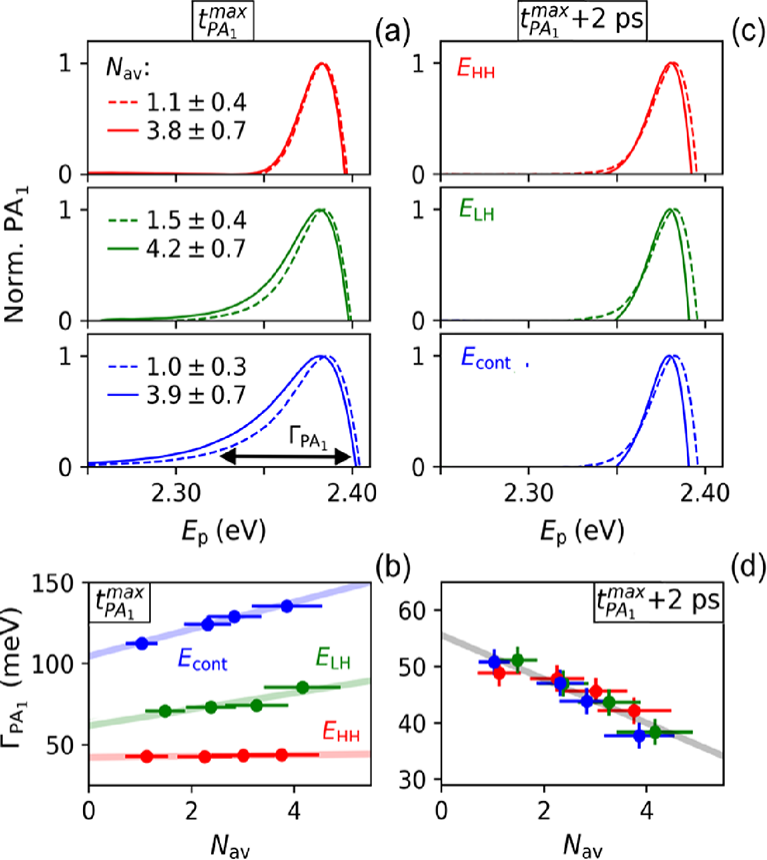
Effects of pump photon energy and *N*_av_ on the PA_1_ feature for *E*_pump_ = *E*_HH_ (red), *E*_LH_ (green), and *E*_cont_ (blue). (a) Normalized PA_1_ features at *t* = *t*_PA_1__^max^ for different *E*_pump_ for low and high average numbers of absorbed photons per NPL (*N*_av_) as indicated. The black thick arrow in the bottom panel indicates the broadening Γ_PA_1__, which is taken as the width at 5% of the PA_1_ magnitude. (b) Effect of *N*_av_ on Γ_PA_1__ at *t* = *t*_PA_1__^max^. (c) PA_1_ features after thermalization at *t* = *t*_PA_1__^max^ + 2 ps. (d) Γ_PA_1__ as a function of *N*_av_ after thermalization.

To further establish the effects of the presence of charge carriers on the TA spectra, we studied the PA_1_ feature for different pump photon energies at short time *t* = *t*_PA_1__^max^ prior to the thermalization of excitons and charge carriers and, after that, at *t* = *t*_PA_1__^max^ + 2 ps. Figure 3a,c shows the PA_1_ features at these times for the lowest and highest values of *N*_av_ in the experiments. At short time and *E*_pump_ = *E*_HH_, the shape of the PA_1_ feature is independent of *N*_av_ (see the top panel in Figure 3a). Apparently, for *N*_av_ values up to about 4, mainly separate free excitons are left after the pump pulse, since the presence of biexcitons (or larger exciton complexes) would cause the probe to produce triexcitons, etc., and a concomitant change of the PA_1_ feature. It appears that for the *N*_av_ values in Figure 3, the population of biexcitons and larger complexes is insignificant, in agreement with the experimental threshold density for gain from biexcitons and with theory.^4,5,14,23^ Interestingly, for *E*_pump_ = *E*_LH_, the PA_1_ feature has an enhanced tail to lower energy with a magnitude that increases with *N*_av_ (middle panel in Figure 3a), which is even more pronounced for *E*_pump_ = *E*_cont_ (see the bottom panel in Figure 3a). We attribute the tail to formation trions, since the number of charge carriers at short time increases with *E*_pump_. This is further demonstrated by considering the energy width, Γ_PA_1__, for which the PA_1_ feature has a magnitude equal to 5% of its maximum (see the black arrow in the bottom panel of Figure 3a). Figure 3b shows that Γ_PA_1__ at *t* = *t*_PA_1__^max^ is independent of *N*_av_ for *E*_pump_ = *E*_HH_ when, initially, only excitons are present. Apparently, exciton–exciton collisions do not cause significant broadening. By contrast, Γ_PA_1__ increases with *N*_av_ for the higher pump photon energies due to the increase in initial charge carrier photogeneration quantum yield. Figure 3c shows that after thermalization at *t* = *t*_PA_1__^max^ + 2 ps, the PA_1_ feature is the same for all pump photon energies, as expected. Interestingly, after thermalization, the width of the PA_1_ feature decreases with *N*_av_ (see Figure 3d). For higher *N*_av_, the quantum yield of excitons increases as compared to charge carriers, since electron–hole recombination becomes more likely.^23^ The decrease in Γ_PA_1__ with *N*_av_ then implies that excitons cause less broadening than charges. In addition, screening effects will decrease when less charges are present.

In summary, the absorbance features PA_1_ and PA_2_ can be assigned to generation of biexcitons and trions by the probe pulse, as discussed further below and in Sections S6 and S7.

### Theoretical Model of Contributions of Excitons, Charge Carriers, Biexcitons, and Trions to the TA Spectra

In Sections S3–S6, we present a theoretical model to describe the contributions of excitons, electrons, holes, biexcitons, and trions to the TA spectra. Here, we summarize the derivation of the expression for the time-dependent absorbance, *A*_on_(*E*_p_, *t*), as a function of probe photon energy *E*_p_ and pump-probe delay time *t*. The result is given in eq 3, which is the same as eq S45 in Section S7.

The bosonic character of photons must of course be properly taken into account to describe their absorption and emission by NPLs. Excitons can to a good approximation also be treated as bosons when their mutual distances exceed the exciton Bohr radius.^24,25^ The latter is the case in the current study, since for the average number of excitons per NPL (*N*_av_ = 3) and the lateral sizes of the NPLs, the average distance between excitons is 8 nm, which is much larger than the 2D exciton Bohr radius of 2 nm.^26^ When *N*_γ_ photons interact with an NPL already containing *N*_X_ excitons, the rate of absorption is then proportional to the factor *N*_γ_(*N*_X_ + 1). The Bose-enhanced absorption gives rise to the factor +*N*_γ_*N*_X_, which is due to the bosonic nature of excitons and takes into account that the rate for creation of an additional exciton is affected by the presence of other excitons around it, as discussed previously for excitons^25^ and exciton-polaritons.^27,28^ Analogously, the rate for emission (EM) of a photon is proportional to *N*_X_(*N*_γ_ + 1) = *N*_X_*N*_γ_ + *N*_X_, where the term *N*_X_*N*_γ_ corresponds to stimulated emission and *N*_X_ to spontaneous emission. Using Fermi’s golden rule,^29^ the net rate for photon absorption is the difference of these two rates, given by
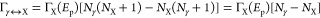
1where the factor Γ_X_(*E*_p_) accounts for the optical absorption cross section and density of final states at probe photon energy *E*_p_ (see Section S3). In eq 1, the Bose-enhanced absorption, proportional to +*N*_γ_*N*_X_, exactly cancels the stimulated emission, proportional to −*N*_X_*N*_γ_, resulting in the factor *N*_γ_ – *N*_X_ in the second right-hand side of eq 1. Hence, the term −*N*_X_ in the latter factor is due to spontaneous photon emission from excitons.

Electrons and holes must on the other hand be described as fermions, as outlined in Section S5. The rate of photon absorption is thus proportional to *N*_γ_*f*_v_(*k*)(1 – *f*_c_(*k*)), with *f*_v_(*k*) being the probability that an electron occupies an HH or LH valence band state with wave vector **k** and *f*_c_(*k*) being the probability that an electron occupies a conduction band state with the same wave vector at energy *E*_p_ above the valence band state. The factor 1 – *f*_c_(*k*) takes into account Pauli blocking by an electron in the conduction band. Similarly, the rate for photon emission by electron–hole recombination is proportional to *f*_c_(*k*)(1 – *f*_v_(*k*))(*N*_γ_ + 1). The first and second terms in the factor *N*_γ_ + 1 bring into account stimulated and spontaneous emission, respectively. The net rate for photon absorption for the single transition considered above becomes (see eqs S24 and S25 for details about the final expression including summation over *k* values and angular momentum quantum numbers of holes and electrons)
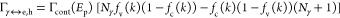
2where Γ_cont_(*E_p_*) is analogous to the factor Γ_X_(*E*_p_) for excitons introduced above. For comparison with experiments, the rates in eqs 1 and 2 must be averaged over the Poisson distribution of the number of probe photons (*N*_γ_) interacting with an NPL, the thermal distribution of excitons over COM motion states (Section S4), the distribution of electrons and holes over *k* states in the valence and conduction bands (Section S5), and the NPL size distribution and disorder (Section S5). Also, eq 2 is averaged over *k*, and in addition, selection rules involving conservation of angular momentum for photoexcitation from the HH and LH valence bands to the conduction band are taken into account (see Section S5).

We describe the TA due to HH-HH and LH-HH biexcitons and trions by two Gaussian functions, *G_i_*(*E*_p_) (see Section S6).

The experimental TA after the pump laser pulse is obtained from *A*_on_(*E*_p_, *t*) = *A*_0_(*E*_p_) + Δ*A*(*E*_p_, *t*) (see Experimental Section). Using this, we get theoretically (eq S45)
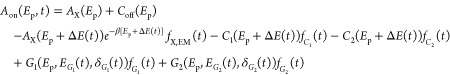
3

The first two terms in eq 3 describe the steady-state absorbance (Figure 1), with photoexcitation to HH and LH exciton states taken into account by  and HH continuum states by *C*_off_(*E*_p_), as reported before.^15^ The third term containing the factor −*A*_X_(*E*_p_ + Δ*E*(*t*)) describes bleach due to (spontaneous) photon emission from excitons. The shape of this spectrum is identical to that in the ground state () but is shifted by a time-dependent energy Δ*E*(*t*) > 0. This energy shift can be due to Coulomb screening by free charges or filling of charge traps. The electrostatic field due to an empty trap reduces the energy of excitons and charge carriers. After filling of the trap by a charge, this field is reduced and the energy for formation of new excitons and charge carriers by the probe increases. The factor exp[ – β(*E*_p_ + Δ*E*(*t*))], where β = 1/*k*_B_*T*, takes into account the Boltzmann distribution of excitons over COM motion energies. The formation and decay kinetics of excitons is described by the factor *f*_X, EM_(*t*) ∝ *N*_X_(*t*) (see eqs S16 and S17). The fourth term containing the factor *C*_1_ describes bleach due to Pauli blocking by charge carriers in the valence and conduction bands, as well as electron–hole recombination by stimulated emission (see Section S5). The fifth term with the factor *C*_2_ takes into account (spontaneous) radiative recombination of electrons and holes. These terms have the same spectral shape as obtained previously^15^ from the steady-state absorbance spectrum in Figure 1, but they are shifted by the same energy, Δ*E*(*t*), as for excitons. The factors *f_C_i__*(*t*) take into account the decay of charge carriers (see eqs S34 and S39). The sixth term with the Gaussian function *G*_1_(*E*_p_, *E*_*G*_1__(*t*), δ_*G*_1__(*t*)) describes the PA_1_ absorption feature in Figure 2b. This feature in the TA is due to formation of an HH-HH biexciton or a trion by the probe pulse, as discussed above and schematically depicted in Figure 2e,f. The peak energy, *E*_*G*_1__(*t*), and standard deviation, δ_*G*_1__(*t*), depend on time as a result of the change of the population of excitons and charge carriers left after the pump pulse and, in turn, the probability that a biexciton or trion is produced by the probe pulse. The formation and decay of excitons and charge carriers are taken into account phenomenologically by the factor *f*_*G*_1__(*t*). The last term containing the Gaussian function *G*_2_(*E*_p_, *E*_*G*_2__(*t*), δ_*G*_2__(*t*)) describes the TA feature PA_2_, which contains possible contributions of a high-energy absorbance tail due to formation of an HH-HH biexciton by the probe pulse and formation of an LH-HH biexciton, as discussed above.

The adjustable fit parameters are specified in Table S1, and the results of the fits are shown in Figure 5 and Figure S4. In the fits, the spectral functions *A_i_*(*E*_p_), *C_i_*(*E*_p_), and *G_i_*(*E*_p_) are normalized with respect to integration over the probe photon energy *E*_p_.

### Theoretical Analysis of the Transient Spectra at Ultrashort Time

**Figure 4:**
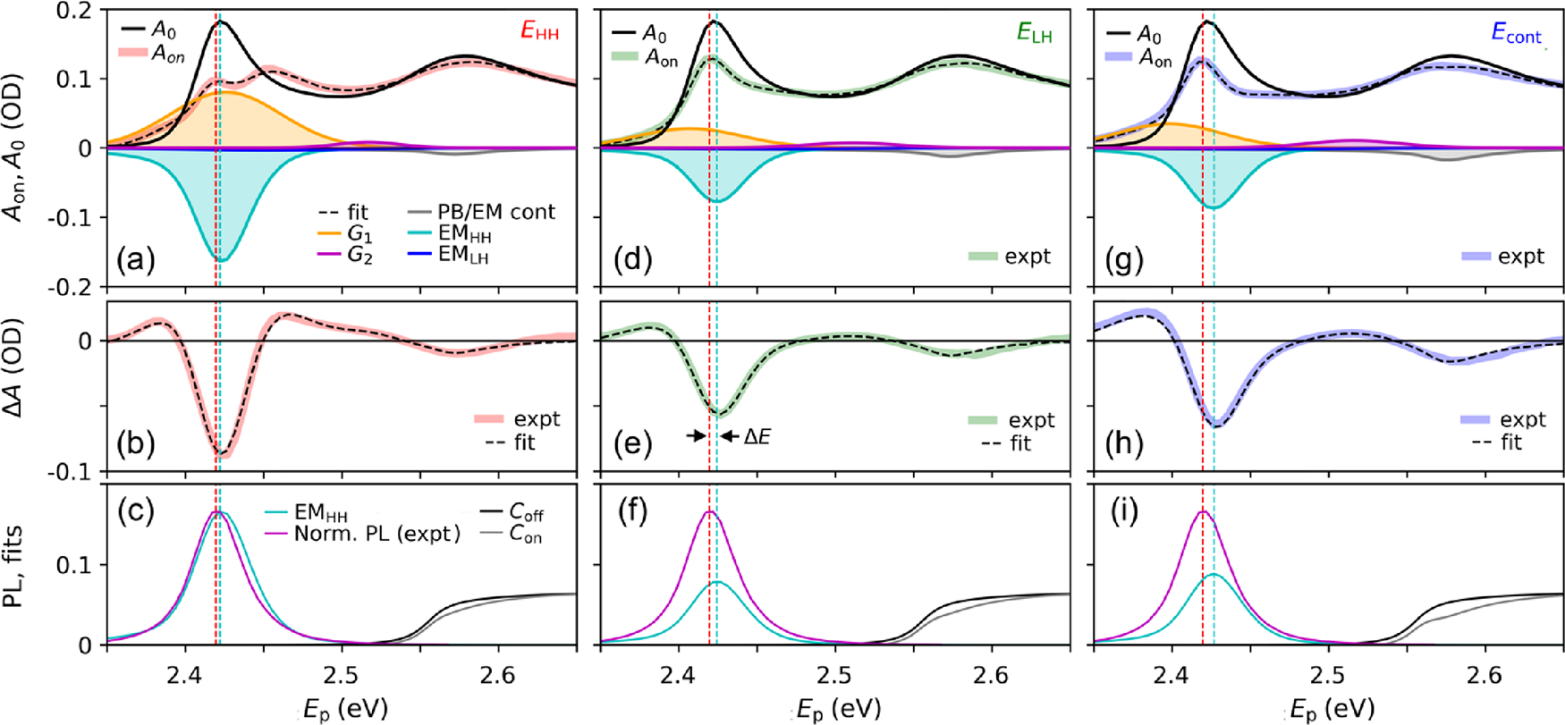
Top row: steady-state absorbance *A*_0_ from Figure 1 and the absorbance *A*_on_(*E*_p_, *t*_PA_1__^max^) at short time, *t*_PA_1__^max^, for *E*_pump_ = *E*_HH_ (red, left column), *E*_LH_ (green, middle column), and *E*_cont_ (blue, right column). The dashed black line shows fits of eq 3 to the *A*_on_ spectra. Contributions of different processes obtained from the fits are also shown: emission from HH excitons (EM_HH_, filled cyan curve), emission from LH excitons (EM_LH_, blue), bleach by Pauli blocking and photon emission (PB/EM) involving charge carriers in continuum states (gray), and PA_1,2_ absorbance features *G*_1_ (yellow) and *G*_2_ (magenta). Middle row: experimental Δ*A*(*E*_p_, *t*_PA_1__^max^) (colored thick lines, same data as in Figure 2b for *t* = *t*_PA_1__^max^) and fits (dashed black lines) with the same parameters as in the top panels. Bottom row: comparison of the fitted transient HH emission (EM_HH_, cyan curve) and steady-state photoluminescence (PL, magenta). Also, the continuum contributions to the steady-state and *A*_on_ spectra are shown as black and gray curves, respectively. The vertical magenta dashed lines indicate the energy at which the PL peak is the maximum, and the vertical cyan dashed lines mark that of the fitted emission from HH excitons (EM_HH_).

The experimental *A*_on_(*E*_p_, *t*_PA_1__^max^) spectra are shown in the top panels of Figure 4 for pump photon energies, *E*_pump_, as indicated. Similar to Figure 2b, the time *t* = *t*_PA_1__^max^ is the time at which the PA_1_ feature reaches its maximum amplitude. The fit of eq 3 to the experimental spectra (black dashed curves) reproduces the measured *A*_on_ spectra extremely well and, consequently, also the Δ*A* spectra in the second row of Figure 4. Figure S3 shows that our model also describes the evolution of the spectra from a subpicosecond timescale up to a long time equal to 1 ns.

Mutual comparison of the top panels in Figure 4 shows that the fitted emission, EM_HH_, from HH excitons (filled cyan curves) and the absorbance described by the Gaussian *G*_1_ (filled yellow curves) for resonant excitation with *E*_pump_ = *E*_HH_ are larger than for the higher pump photon energies. The larger amount of HH excitons produced initially in the case of *E*_pump_ = *E*_HH_ leads to the strongest emission and also to the most significant contribution of HH-HH biexciton formation by the probe pulse. The bleach due to emission from HH excitons is superposed on the broad absorbance *G*_1_, and together, this results in the appearance of two local maxima in the *A*_on_ spectrum for *E*_pump_ = *E*_HH_. The *A*_on_ and Δ*A* spectra exhibit a low energy absorption tail that becomes more significant as *E*_pump_ goes up and causes the maximum of the fitted *G*_1_ to shift to lower energy. The tail is in part due to the broadening of the HH exciton peak, which will increase at higher *E*_pump_ when more charge carriers are generated and exciton–charge interactions become more important.^20^

The contribution of the Gaussian *G*_2_ (magenta) to the *A*_on_ spectra is much smaller than that of *G*_1_. From this, we infer that the broadening of the HH exciton peak occurs mainly to lower energy and formation of LH-HH biexcitons (at energy above the HH exciton peak) by the probe pulse is less significant than formation of HH-HH excitons. Apparently, the interaction between an LH exciton and an HH exciton is smaller than between two HH excitons. For *E*_pump_ = *E*_cont_, the magnitude of *G*_2_ is slightly larger than for lower pump photon energies. This can be due to formation of more trions, where the probe pulse produces an LH exciton near a charge carrier produced by the pump pulse.

The fitted bleach due to LH excitons (EM_LH_, blue curves) is negligible for all pump photon energies (consequently, the blue curve EM_LH_ is hardly observable in Figure 4). Since this is even the case for *E*_pump_ = *E*_LH_, thermal relaxation of LH excitons to HH excitons and/or dissociation into electrons and holes in continuum states occurs within the timescale *t*_PA_1__^max^, which is a few hundred femtoseconds (see Figure S1).

**Figure 5:**
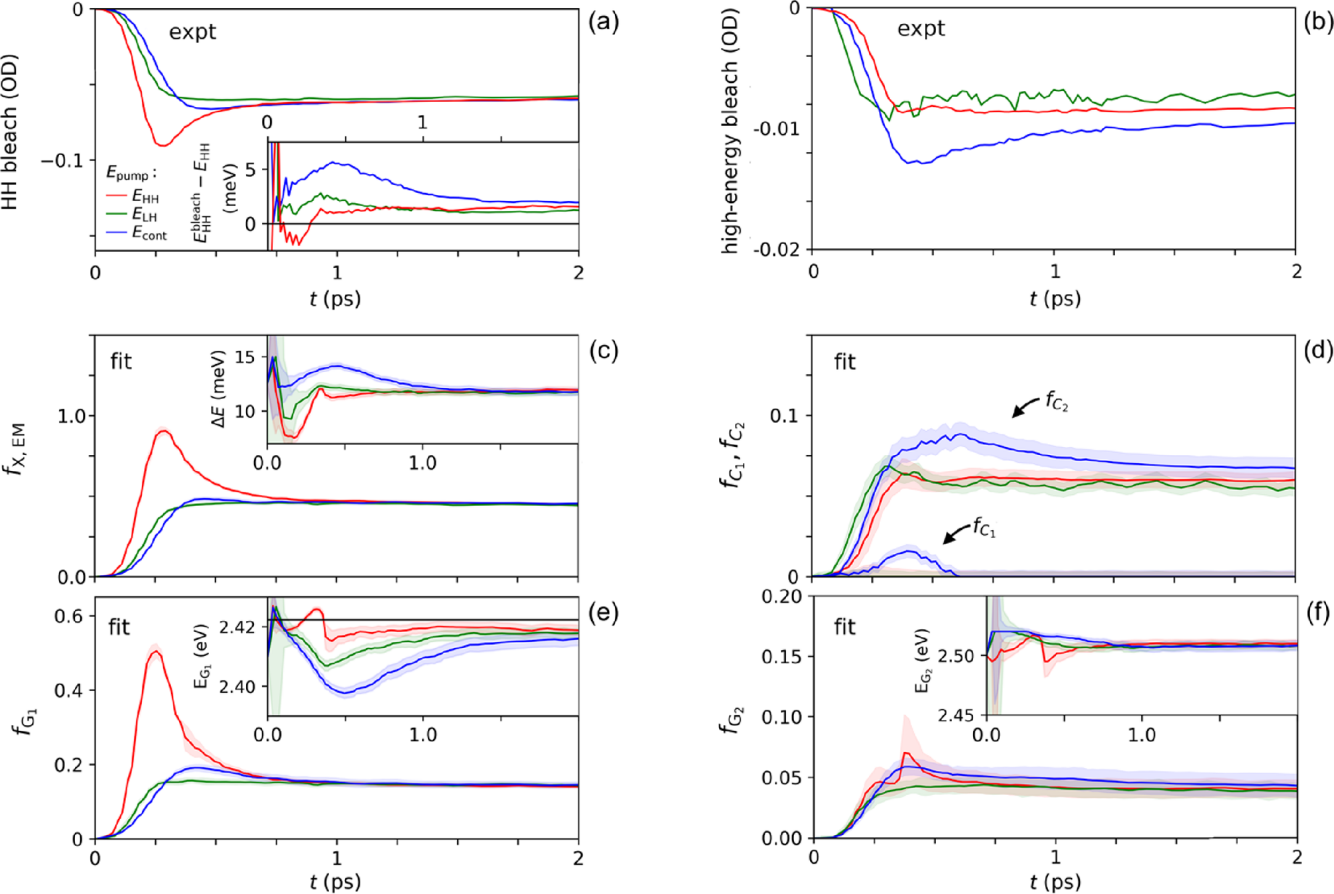
(a) Kinetics of the experimental HH bleach amplitude after different excitation energies, *E*_pump_. The inset shows the energy difference between the energy at which the HH bleach has the largest amplitude, *E*_HH_^bleach^, and the energy of the HH peak in the absorbance spectrum in Figure 1. (b) Kinetics of the experimental high-energy bleach amplitude. (c–f) Coefficients *f_i_* and energies obtained from fits of eq 3 to the experimental spectra in the top panels in Figure S3. The horizontal black line in the inset of panel (e) represents the energy *E*_HH_ of the first exciton peak in the absorbance spectrum in Figure 1. The shaded areas represent the uncertainties of the fit parameters.

The magenta curves in the bottom panels of Figure 4 show the steady-state photoluminescence (PL) spectrum of the NPLs from our previous study.^15^ The vertical magenta dashed lines indicate the energy at which the PL peak is the maximum. The vertical cyan dashed lines mark the energy of the fitted bleach, EM_HH_, at *t* = *t*_PA_1__^max^ in the TA spectra. The energy EM_HH_ increases with pump photon energy, which is reflected in the increase in the fitted value of Δ*E*(*t* = *t*_PA_1__^max^) from about 8 meV to almost 15 meV in eq 3 (see Figure 5c and Figure S4). On short times, more charge carriers will be present at higher pump photon energy, resulting in enhanced Coulomb screening and a concomitant increase in the exciton energy and, thus, EM_HH_. This blue shift can also result from filling of a charge trap, which causes an increase in the energy of the remaining excitons in the NPL that are not trapped. Quenching of excitons by charge trapping has been found to be significant in CdSe NPLs^8−11,13,19,30^ and will contribute to the blue shift of EM_HH_ in Figure 4 with respect to the PL maximum. Since even for *E*_pump_ = *E*_HH_, there is a blue shift, dissociation of HH excitons into free charge carriers and/or trap filling already occurs on the few hundred femtosecond timescale of *t*_PA_1__^max^. Such ultrafast trap filling by hole transfer from HH excitons has been reported previously for CdSe NPLs.^10^

The top panels in Figure 4 show that the magnitude of the fitted bleach by charge carriers due to Pauli blocking and photon emission (PB/EM, gray curves) at higher probe energy (near 2.58 eV) increases with pump photon energy. This reflects a larger initial yield of charge carriers for higher pump photon energy. The effect of bleach due to charge carriers is further illustrated in the bottom panels of Figure 4, where we show the fitted steady-state absorbance, *C*_off_ (red dashed curve in Figure 1), together with the fitted result at time *t*_PA_1__^max^ after the pump pulse, which is equal to *C*_on_ = *C*_1_(*E*_p_ + Δ*E*(*t*_PA_1__^max^))*f*_*C*_1__(*t*_PA_1__^max^) – *C*_2_(*E*_p_ + Δ*E*(*t*_PA_1__^max^))*f*_*C*_2__(*t*_PA_1__^max^) in eq 3. The decrease in the magnitude of *C*_on_ with pump photon energy is clearly observable.

### Theoretical Analysis of Exciton and Charge Carrier Dynamics

Below, we discuss the dynamics of formation and relaxation of excitons and charge carriers. Data at short time until 2 ps are shown in Figure 5, and results on longer times up to 2 ns are presented in Figures S3 and S4. Here, *t* = 0 is the time at which the measured Δ*A* signal is above the experimental noise level.

Figure 5a shows the measured kinetics of the amplitude of the HH bleach peak. It rises most rapidly for a pump photon energy equal to *E*_HH_ and becomes slower on increasing *E*_pump_. This reflects the slower population of HH excitons by thermal relaxation of initially more energetic holes and electrons at higher *E*_pump_. A pronounced decay is seen after *E*_HH_ excitation, similar to results of Cassette et al. who attributed this to exciton quenching by hole trapping.^10^ The absence of this decay at higher pump photon energies implies that the rate of charge trapping increases with energy. The inset in Figure 5a shows the experimental energy shift of the HH bleach magnitude from the steady-state HH absorbance peak in Figure 1. The blue shift is more pronounced for higher pump photon energy, which can be assigned to the larger number of charge carriers increasing the exciton energy by Coulomb screening. The fitted coefficient *f*_X, EM_(*t*) and energy shift Δ*E*(*t*) in Figure 5c resemble these effects in the experimental data in Figure 5a, as expected. These fit parameters are independent of pump photon energy after 1 ps when excitons and charge carriers have thermalized. The magnitude of *f*_X, EM_(*t*) decreases on a nanosecond timescale due to the decay of excitons to the ground state, while the energy shift remains virtually constant (see Figure S4). The constant energy shift agrees with slow recombination of trapped holes with electrons on a timescale of the order of 100 ns.^9,30^

The experimental kinetics of the high-energy bleach amplitude due to charge carriers in valence and conduction band states is shown in Figure 5b. After a few hundred femtoseconds, the amplitude becomes the largest for the highest pump photon energy, *E*_cont_, which reflects that for this energy, only charge carriers are produced initially. The fitted coefficients *f*_*C*_1__(*t*) and *f*_*C*_2__(*t*) reflect the experimental trend and converge to similar magnitude within 2 ps. The rise time of *f*_*C*_2__(*t*) is the longest for *E*_HH_ excitation, since initially, only HH excitons are formed that must dissociate into charge carriers to contribute to the high-energy bleach. For *E*_LH_ and *E*_cont_ excitations, *f*_*C*_2__(*t*) increases on a similar timescale, owing to fast dissociation of LH excitons into charge carriers. Figure S4 shows that the decrease in *f*_*C*_2__(*t*) occurs with the same kinetics as *f*_X, EM_(*t*), resembling charge recombination to the ground state. The coefficient *f*_*C*_2__(*t*) is much larger than *f*_*C*_1__(*t*), which implies that the high-energy bleach is mainly due to spontaneous photon emission via electron–hole recombination, rather than stimulated emission or Pauli blocking.

The time dependence of the coefficient *f*_*G*_1__(*t*) in Figure 5e describing formation of biexcitons and trions by the probe pulse is similar to that of *f*_X, EM_(*t*), which reflects the exciton population left after the pump pulse. From this resemblance, we infer that the function *G*_1_ is largely due to biexciton formation and, to a smaller extent, to trion formation. The inset of Figure 5e shows that around 0.5 ps, the value of *E*_*G*_1__ decreases when the pump photon energy becomes higher. A larger initial amount of charge carriers generated at higher pump photon energy causes enhanced broadening^20^ of the HH exciton, which can give rise to a lower *E*_*G*_1__ from the fit. The coefficient *f*_*G*_2__ and energy *E*_*G*_2__ in Figure 5f for *E*_cont_ excitation are initially slightly larger than for lower pump photon energies. As mentioned in Theoretical Analysis of the Transient Spectra at Ultrashort Time, this could be associated with the probe pulse producing trions consisting of an LH exciton near a charge carrier left after the pump pulse.

## Conclusions

The initial photogeneration quantum yields of charges versus excitons in CdSe NPLs increase with photon energy. After 1 ps, excitons and charges have relaxed to the same energy distribution, irrespective of the pump photon energy. The TA spectra could be described very accurately by a theoretical model that treats photons and excitons as bosons, while electrons and holes are treated as fermions. For the highest number of about four absorbed photons per NPL in our experiments, excitons are behaving independently from each other without observable signatures of the presence of biexcitons or larger complexes. Biexcitons and trions were observed due to formation of an exciton by a probe photon near an already present exciton or charge carrier left after the pump pulse. We observed a TA signal at the high-energy side of the HH absorption peak, which can result from formation of trions consisting of a charge and an LH exciton or LH-HH biexcitons. The theoretical model presented in detail in the Supporting Information is generally applicable to interpretation of TA data on two-dimensional materials.

## Supporting Information Available

Determination of the average number of absorbed pump photons by an NPL, definition of transient optical absorbance (TA), TA kinetics on long timescale, theoretical description of the TA signal including contributions of excitons, charge carriers, biexcitons, and trions, and effects of exciton center-of-mass motion, nanoplatelet size distribution, and disorder (PDF)
