# Supplementary material for: Effects of Pump Photon Energy on Generation and Ultrafast Relaxation of Excitons and Charge Carriers in CdSe Nanoplatelets
Source: J Phys Chem C Nanomater Interfaces. 2023 Jan 19;127(4):1899–907. doi: 10.1021/acs.jpcc.2c07292 (PMC9900632; doi:10.1021/acs.jpcc.2c07292)
Supplement: Supplementary file 1 — jp2c07292_si_001.pdf [file jp2c07292_si_001.pdf]

Supporting Information

Effects of Pump Photon Energy on Generation and Ultrafast Relaxation of Excitons and Charge Carriers in CdSe Nanoplatelets

Michele Failla,[1] Fransisco García Flórez,[2] Bastiaan B. V. Salzmann,[3] Daniel Vanmaekelbergh,[3] Henk T. C. Stoof,[2] and Laurens D. A. Siebbeles[1]

1. Chemical Engineering Department, Delft University of Technology, Van der Maasweg 9, 2629 HZ Delft, The Netherlands

2. Institute for Theoretical Physics and Center for Extreme Matter and Emergent Phenomena, Utrecht University, Princetonplein 5, 3584 CC Utrecht, The Netherlands

3. Debye Institute, Condensed Matter and Interfaces, Utrecht University, Princetonplein 1, 3584 CC Utrecht, The Netherlands

S1. TA kinetics and determination of the average number of absorbed photons per nanoplatelet

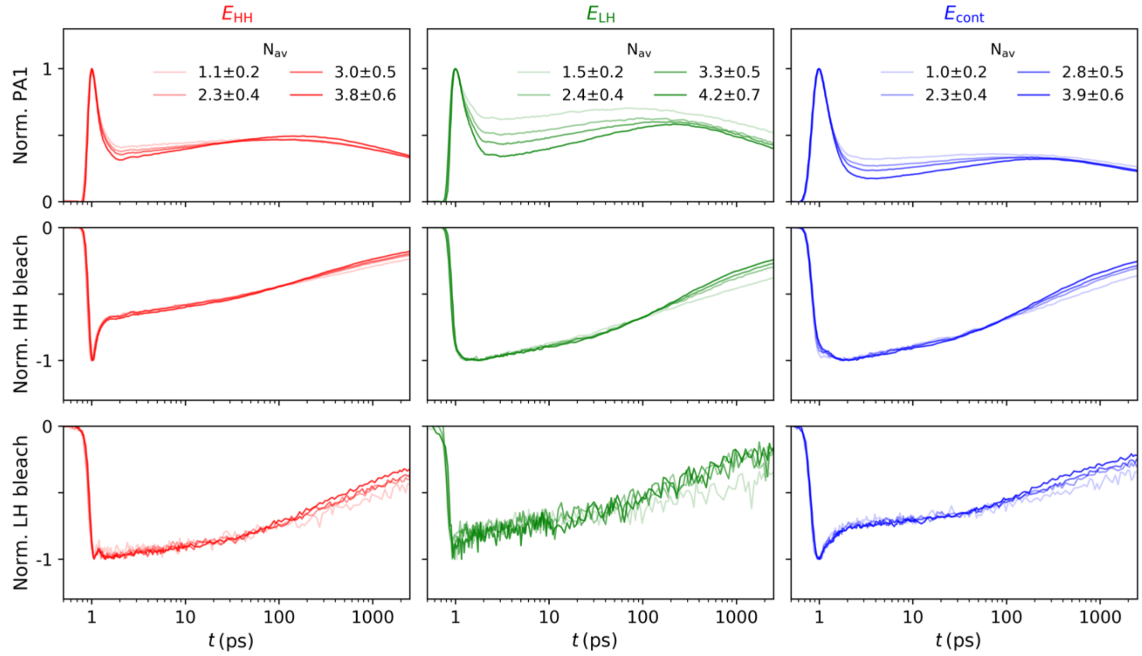

Figure S1. Decay kinetics of TA traces normalized at maximum amplitude after excitation with a pump photon energy equal to E_{HH} (first column, red), E_{LH} (middle column, green) and E_{cont} (right column, blue). The probe photon energy was taken at the maximum of the PA1 feature (top row), the HH bleach (middle row) and at the high-energy bleach feature around $E_{LH} =$ 2.58 eV (bottom row). The probe photon energies varies during about 1 ps after the maximum TA amplitude has been reached, due to the change of the shape of the TA spectra on this timescale, as discussed in the main text. Traces are shown for different average numbers of absorbed pump photons per nanoplatelet, N_{av}, which were determined as described below.

The average number of absorbed pump photons with energy E_{pump} by a NPL is equal to

$$N_{\text{av}}(E_{pump}) = I_0(E_{pump})\,\mu(E_{pump})\,V_{NPL}, \tag{S1}$$

with $I_0(E_{pump})$ the laser fluence in a pump pulse, $\mu(E_{pump})$ the absorption coefficient of a NPL with volume V_{NPL}. The incident fluence was calculated according to

$$I_0(E_{pump}) = \frac{P_{pump}}{E_{pump}\, f_{pump}\, \pi r^2}\ , \tag{S2}$$

where P_{pump} is the pump power measured using a pinhole with radius $r = 1$ mm, and f_{pump} = 2500 Hz is the frequency of pump pulses arriving at the sample. The uncertainty of P_{pump} is about 0.1 mW, which stems from the laser instability during time and inaccuracy of the overlap between the pump and probe laser pulses in the sample. Values of $\mu(E_{pump})$ were obtained as described previously.[1] For photoexcitation to the HH exciton at 2.42 eV, the LH exciton at 2.58eV and into the continuum at 3.10 eV this gives, $\mu = 3.25 \times 10^6$ cm^{-1}, $\mu = 2.36 \times 10^6$ cm^{-1}and $\mu = 1.80 \times 10^6$ cm^{-1}, respectively. The volume, V_{NPL}, of the NPLs was calculated from their lateral sizes ($L_x = 25 \pm 3$ nm, $L_y = 8 \pm 1$ nm) and their width of 4.5 monolayers ($L_z = 1.37$ nm) yielding $V_{NPL} = L_x L_y L_z = 280 \pm 45$ nm^3.[2] Figure S1 shows the measured HH bleach amplitude 2 ps after

photoexcitation to the HH, LH excitons and into the continuum at 3.10 eV, as a function of N_{av}. As expected, the HH bleach depends linearly on N_{av}. The uncertainty in the values of N_{av} reflects that of P_{pump} and V_{NPL} in Equations (S1) and (S2).

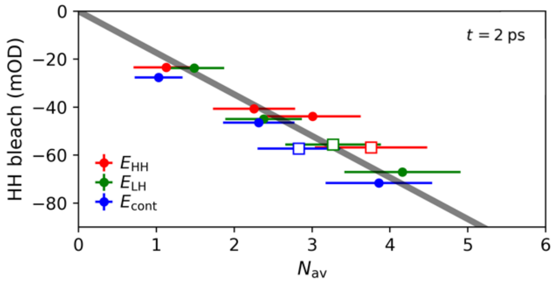

Figure S2. HH bleach amplitude as a function of the average number of absorbed photons per NPL (N_{av}) at 2 ps after photoexcitation with $E_{pump} = E_{HH}$ (red dots), E_{LH} (green) and E_{cont} (blue). The empty squares correspond to the values of N_{av} during the experiments to obtain the data shown in Figures 2, 4 and 5 of the main text (i.e. $N_{\mathrm{av}} = 3.8 \pm 0.6$, $N_{\mathrm{av}} = 3.3 \pm 0.5$ and $N_{\mathrm{av}} = 2.8 \pm 0.5$ for E_{HH}, E_{LH} and E_{cont}, respectively). The grey line is a guide to the eye highlighting the linear dependence of the bleach on N_{av}.

S2. Definition of transient optical absorbance

Without the pump laser pulse the absorbance $A_{off}(\hbar\omega)$ at probe photon energy $\hbar\omega$ due to NPLs with optical absorption cross section $\sigma(\hbar\omega)$ and volume density n_{NPL} in a cuvette with optical path length d is defined via the relation

$$I_{off}(\hbar\omega) = I_0(\hbar\omega)\, 10^{-A_{off}(\hbar\omega)} = I_0(\hbar\omega)\, e^{-\sigma(\hbar\omega) n_{\mathrm{NPL}} d}, \tag{S3}$$

with $I_0(\hbar\omega)$ and $I_{off}(\hbar\omega)$ being the incident and transmitted probe pulse fluencies. After the pump pulse, the presence of excitons (X) and free electrons (e) and holes (h) causes the absorbance to change to $A_{on}(\hbar\omega)$ and the transmitted probe fluence becomes

$$I_{on}(\hbar\omega) = I_0(\hbar\omega)\, 10^{-A_{on}(\hbar\omega)} \equiv I_0(\hbar\omega)\, e^{-S_{on}(\hbar\omega)}, \tag{S4}$$

with the optical absorption signal after the pump pulse defined as

$$S_{on}(\hbar\omega) = A_{on}(\hbar\omega)\, ln(10). \tag{S5}$$

Our transient absorption setup provides the difference $\Delta A(\hbar\omega) = A_{on}(\hbar\omega) - A_{off}(\hbar\omega)$. We obtain $A_{on}(\hbar\omega)$ by separately measuring the steady-state optical absorbance $A_0(\hbar\omega)$ of the NPL solution (which corresponds to $A_{off}(\hbar\omega)$ in the transient optical absorption measurements). Hence, we use $A_{on}(\hbar\omega) = \Delta A(\hbar\omega) + A_0(\hbar\omega)$.

The optical absorption signal after the pump pulse is affected by the presence of excitons and charge carriers, which we describe according to

$$S_{on}(\hbar\omega) = \left[\sigma_X(\hbar\omega, \bar{N}_X)[1 - f_X(\hbar\omega, \bar{N}_X)] + \sigma_{\text{cont}}(\hbar\omega, \bar{N}_e, \bar{N}_h)[1 - f_{\text{cont}}(\hbar\omega, \bar{N}_e, \bar{N}_h)]\right] n_{\text{NPL}} d, \quad \text{(S6)}$$

with $\bar{N}_X, \bar{N}_e$ and $, \bar{N}_h$ the average number of excitons, free electrons and holes in a NPL, respectively. The factors f_X and f_{cont} take into account the enhanced transmitted fluence of probe light due to (probe-pulse induced) photon emission from excitons and Pauli blocking by charge carriers, respectively. Note, that in Equation (S6) we make the approximation that the cross sections depend on the average number of excitons and charge carriers rather than their actual number. As discussed in Section S6, the occurrence of quenching of excitons by charge trapping causes the cross sections of excitons and remaining charge carriers to shift to higher energy. In the absence of photoexcitation of the sample by the pump pulse we have $N_X = N_e = N_h = 0$, and $f_X = f_{cont} = 0$, so that Equation (S4) reduces to the ground state absorption spectrum $S_{off}(\hbar\omega) = A_{off}(\hbar\omega)\, ln(10) = [\sigma_X(\hbar\omega) + \sigma_{\text{cont}}(\hbar\omega)] n_{\text{NPL}} d.$ Note, that σ_X and σ_{cont} depend on the center-of-mass (COM) motion of excitons, which is determined by the lateral confinement in the NPLs, as discussed in Section S4 and in our previous work.[2] We also take into account that the measured optical absorption, $S_{on}(\hbar\omega)$, depends on the distribution of excitons and charge carriers over (thermal) energy ($k_B T$), see Sections S3 - S5.

S3. Transient absorption due to excitons

According to the Fermi's golden rule, the rate for an optical transition of an electron between initial (i) and final (f) states is given by[3]

$$\Gamma_{i \to f} = \frac{2\pi}{\hbar} |\langle i|H_{int}|f\rangle|^2 \, \rho_X(\hbar\omega), \quad \text{(S7)}$$

where the perturbation H_{int} describes the dipolar interaction between the electron and the electric field due to photons and $\rho_X(\hbar\omega)$ is the density of exciton states at photon energy $\hbar\omega$ above the ground state. Photons are true bosons, while excitons can to a good approximation be treated as bosons provided their mutual distances are much longer than their Bohr radius.[4-5] Taking into account the bosonic nature of these species, the average rate of photon absorption over all NPLs with initial states $|N_X, N_\gamma\rangle$, of N_X excitons and interacting with N_γ probe photons, to a final state $|N_X + 1, N_\gamma - 1\rangle$ is

$$\langle\, \Gamma_{\gamma \to X}\, \rangle = \Gamma_X(\hbar\omega) \sum_{N_X=0, N_\gamma=0}^{\infty} \left|\langle N_X + 1, N_\gamma - 1|\hat{\Psi}_X^{+}\hat{a}|N_X, N_\gamma\rangle\right|^2 P_{N_X}(\hbar\omega) P_{N_\gamma}(\hbar\omega) = \quad \text{(S8)}$$

$$= \Gamma_X(\hbar\omega) \sum_{N_X,=0, N_\gamma=0}^{\infty} N_\gamma (N_X + 1)\, P_{N_X}(\hbar\omega) P_{N_\gamma}(\hbar\omega)\,.$$

In Equation (S8), $\hat{a}$ is the photon annihilation operator, $\hat{\Psi}_X^{+}$ is the exciton creation operator, $P_{N_X}(\hbar\omega)$ and $P_{N_\gamma}(\hbar\omega)$ are the probabilities of having a NPL with N_X excitons interacting with N_γ photons of energy $\hbar\omega$. The probability P_{N_X} is determined by the product of the number of pump photons absorbed in a NPL (Poisson distribution) and the quantum yield of excitons with energy $\hbar\omega$. The factor $\Gamma_X(\hbar\omega)$ brings into account the optical transition dipole moment and density of final states. We have taken this factor out of the summation, which implies that the optical absorption cross section $\sigma_X(\hbar\omega, \bar{N}_X)$ is assumed to

be determined by the average number ($\bar{N}_X$) of excitons in a NPL, rather than the actual number. Analogous to Equation (S6), the average rate for emission of a photon with an exciton recombining to the ground state is given by

$$\langle \Gamma_{X\to\gamma} \rangle = \Gamma_X(\hbar\omega) \sum_{N_X=1,N_\gamma=0}^{\infty} \left|\langle N_X-1, N_\gamma+1|\hat{\Psi}_X \hat{a}^+|N_X, N_\gamma\rangle\right|^2 P_{N_X}(\hbar\omega)\, P_{N_\gamma}(\hbar\omega) = \tag{S9}$$

$$= \Gamma_X(\hbar\omega) \sum_{N_X=0,N_\gamma=0}^{\infty} N_X\left(N_\gamma+1\right) P_{N_X}(\hbar\omega)\, P_{N_\gamma}(\hbar\omega)\ .$$

The net rate resulting from both photon absorption and emission is then

$$\langle \Gamma_{\gamma\leftrightarrow X} \rangle = \langle \Gamma_{\gamma\to X} \rangle - \langle \Gamma_{X\to\gamma} \rangle$$

$$= \Gamma_X(\hbar\omega) \sum_{N_X,N_\gamma=0}^{\infty} \left[N_\gamma(N_X+1) - N_X\left(N_\gamma+1\right)\right] P_{N_X}(\hbar\omega) P_{N_\gamma}(\hbar\omega) = \tag{S10}$$

$$= \Gamma_X(\hbar\omega) \sum_{N_X,N_\gamma=0}^{\infty} \left[N_\gamma - N_X\right] P_{N_X}(\hbar\omega) P_{N_\gamma}(\hbar\omega) = \Gamma_X(\hbar\omega)\left[\bar{N}_\gamma(\hbar\omega) - \bar{N}_X(\hbar\omega)\right],$$

where we have used

$$\sum_{N_i=0}^{\infty} P_{N_i}(\hbar\omega) = 1 \quad \text{and} \quad \sum_{N_i=0}^{\infty} N_i P_{N_i}(\hbar\omega) = \bar{N}_i(\hbar\omega)\ . \tag{S11}$$

In Equation (S11) and below we explicitly specify the average number of excitons and photons at a particular probe photon energy $\hbar\omega$ by using the notation $\bar{N}_i(\hbar\omega)$. In Equation (S10), the last term is due to spontaneous emission only. The stimulated emission is cancelled by the Bose enhancement of the absorption.

The decay of the average number of probe photons $\bar{N}_\gamma(\hbar\omega, x)$ per unit path length at position x in the sample is

$$\frac{d\bar{N}_\gamma(\hbar\omega, x)}{dx} = -\frac{1}{c}\langle \Gamma_{\gamma\leftrightarrow X} \rangle V\, n_{\mathrm{NPL}}, \tag{S12}$$

with c the velocity of light in the sample and V is the volume encountered by the probe pulse. Since $\bar{N}_\gamma(\hbar\omega, x)$ is directly proportional to the laser probe fluence $I(\hbar\omega, x)$, we can use Equations (S10) and (S12) to get

$$\frac{1}{\bar{N}_\gamma(\hbar\omega,x)}\frac{d\bar{N}_\gamma(\hbar\omega,x)}{dx}=\frac{1}{I(\hbar\omega,x)}\frac{dI(\hbar\omega,x)}{dx}=-\frac{1}{\bar{N}_\gamma(\hbar\omega,x)}\frac{\langle\,\Gamma_{\gamma\leftrightarrow X}\,\rangle}{c}V\,n_{\mathrm{NPL}}$$
$$=\ -\sigma_X(\hbar\omega,\bar{N}_X)\,n_{\mathrm{NPL}}\left[\frac{\bar{N}_\gamma(\hbar\omega,x)-\bar{N}_X(\hbar\omega,x)}{\bar{N}_\gamma(\hbar\omega,x)}\right] \tag{S13}$$
$$=\ -\sigma_X(\hbar\omega,\bar{N}_X)\,n_{\mathrm{NPL}}\left[1-\frac{\bar{N}_X(\hbar\omega,x)}{\bar{N}_\gamma(\hbar\omega,x)}\right],$$

with $\sigma_X(\hbar\omega,\bar{N}_X)=\Gamma_X(\hbar\omega)V/c$ the optical absorption cross section. Note that the average number of excitons, $\bar{N}_X(\hbar\omega,x)$, depends on the position x due to absorption of the pump laser pulse along its path through the sample. Integrating Equation (S13) over the optical path length yields

$$\int_{I(\hbar\omega,0)}^{I(\hbar\omega,d)}\frac{1}{I(\hbar\omega,x)}\,dI(\hbar\omega,x)=\ln\left(\frac{I(\hbar\omega,d)}{I(\hbar\omega,0)}\right)\ =-\sigma_X(\hbar\omega,\bar{N}_X)\,n_{\mathrm{NPL}}\left[d-\int_0^d\left[\frac{\bar{N}_X(\hbar\omega,x)}{\bar{N}_\gamma(\hbar\omega,x)}\right]dx\right] \tag{S14}$$

and the first term in Equation (S6) due to excitons is then found to be

$$S_X(\hbar\omega)=\sigma_X(\hbar\omega,\bar{N}_X)\ \ [1-f_X(\hbar\omega)]\,n_{\mathrm{NPL}}\,d=\sigma_X(\hbar\omega,\bar{N}_X)\,n_{\mathrm{NPL}}d\left[1-\frac{1}{d}\int_0^d\left[\frac{\bar{N}_X(\hbar\omega,x)}{\bar{N}_\gamma(\hbar\omega,x)}\right]dx\right], \tag{S15}$$

so that

$$f_X(\hbar\omega)=\frac{1}{d}\int_0^d\left[\frac{\bar{N}_X(\hbar\omega,x)}{\bar{N}_\gamma(\hbar\omega,x)}\right]dx. \tag{S16}$$

The factor $f_X(\hbar\omega)$ is determined by the bosonic nature of photons and excitons (*i.e.* the terms '+1' in Equation (S10)).

The initially photogenerated excitons or free electrons and holes will relax thermally. We assume this leads to formation of HH excitons and charge carriers. We only consider HH excitons, since the population of excitons with higher energy is negligible, as discussed before.[6] For a Bose-Einstein distribution of HH excitons over COM states their chemical potential, μ_X, is an independent fitting parameter. Fortunately, for the size of our NPLs with each containing at most 4 excitons a Maxwell-Botlzmann distribution is a good approximation, see Figs. 1 and 7 in our previous work.[6] For a Maxwell-Boltzmann distribution $\bar{N}_X(\hbar\omega,x)$ is proportional to a thermal energy distribution factor $e^{-\beta(\hbar\omega-\mu_X)}$, where $\beta=1/k_BT$. Now the factor $e^{-\beta\mu_X}$ can be included in the fitted amplitude of $f_X(\hbar\omega)$, see eq. S17. During time, excitons decay back to the ground state and consequently $\bar{N}_X(\hbar\omega,x)$ decreases with the time delay t between the pump and probe laser pulses. These two effects are taken into account by expressing Equation (S16) as the product of the thermal distribution factor and another factor describing the decay of the total exciton population by photon emission (EM) according to

$$f_X(\hbar\omega)=\ e^{-\beta\hbar\omega}f_{X,\mathrm{EM}}(t). \tag{S17}$$

Comparing Equations (S16) and (S17) shows that $f_{X,\mathrm{EM}}(t)$ is inversely proportional to the photon fluence, $\bar{N}_\gamma(\hbar\omega, x)$, of the probe laser pulse. The latter is due to the bosonic nature of the photons and excitons. To the best of our knowledge, the dependence of the transient optical absorption on the probe fluence has not been explicitly taken into consideration before. Note that Equation (S15) depends on the ratio of the number excitons in a NPL and the number of probe photons arriving in a NPL. For $\bar{N}_X \gg \bar{N}_\gamma$ photon emission dominates the transient absorption spectrum $S_X(\hbar\omega)$, while for $\bar{N}_X \ll \bar{N}_\gamma$ the emission becomes negligible. Finally, the contribution of excitons to S_{on} [first term in Equation (S6)] is found to be

$$S_X(\hbar\omega) = \left[\sigma_X(\hbar\omega, \bar{N}_X)\left[1 - e^{-\beta\hbar\omega} f_{X,\mathrm{EM}}(t)\right]\right] n_{\mathrm{NPL}} d. \tag{S18}$$

As mentioned at the end of Section S2, for comparison with experiments we must average Equation (S18) over the occupation of excitons in states with different COM translational energy and bring into account the lateral size distribution of the NPLs, as well as effects of (in)homogenous broadening. This is described next in Section S4.

S4. Effects of exciton COM energy, NPL size distribution and disorder on transient absorption due to excitons

Similar to our previous work, we write the wave function of an exciton as[2]

$$\Psi_X(z_e, z_h, \mathbf{r}, \mathbf{R}) = u^e_{n_z}(z_e)\, u^h_{n_z}(z_h) \psi^{rel}(\mathbf{r}) \psi^{COM}_{n_x,n_y}(\mathbf{R}). \tag{S19}$$

Here, $z_{e,h}$ is the coordinate of an electron (e) or hole (h) perpendicular to the plane of the NPL, $\mathbf{r} = \mathbf{r}_e - \mathbf{r}_h$ and $\mathbf{R} = (m_e\mathbf{r}_e + m_h\mathbf{r}_h)/(m_e + m_h)$ are the relative and COM coordinates in the plane of the NPL, respectively, with m_e (m_h) the electron (hole) effective mass, and $m_e + m_h = M$ the total exciton mass. The COM motion of excitons in a NPL with lateral sizes $\boldsymbol{L} = (L_x, L_y)$ is described by the particle-in-a-box model with quantum numbers $\boldsymbol{n} = (n_x, n_y)$, yielding energies

$$E^J_{\boldsymbol{n}}(\boldsymbol{L}) = E^J + \frac{\pi^2\hbar^2}{2M^J}\left[\left(\frac{n_y}{L_x}\right)^2 + \left(\frac{n_y}{L_y}\right)^2\right]. \tag{S20}$$

Here, J = HH, LH indicates the heavy and light hole excitons and E^J is the energy of excitons without COM energy. The optical absorption cross section σ^J for excitation from the ground state is proportional to a factor a^J taking into account the relative motion of the electron and hole in an exciton, and also a factor, $f^{osc}_n(\boldsymbol{L})$, in the optical oscillator strength arising from the translational COM motion of the exciton in a NPL, *i.e.* $\sigma^J = a^J f_{\boldsymbol{n}}(\boldsymbol{L})$, with[2]

$$f^{osc}_n(\boldsymbol{L}) = \int_0^{L_x}\int_0^{L_y} dx\, dy\, |\psi^{COM}_{n_x,n_y}(\mathbf{R})|^2 = \frac{16 L_x L_y (1-(-1)^{n_x})(1-(-1)^{n_y})}{n_x^2 n_y^2}. \tag{S21}$$

Note that $f^{osc}_n(\boldsymbol{L})$ is non-zero only if both n_x and n_y are odd. According to Equation (S21) σ_X in Equation (S18) depends on $\boldsymbol{n}$ and $\boldsymbol{L}$. For comparison with experiments, we need to average $S_X(\hbar\omega)$ over the occupation of states with different $\boldsymbol{n}$ and the lateral size distribution $D(\boldsymbol{L})$ of the NPLs. In addition, we take into account the homogeneous broadening due to exciton-

phonon interaction by a Lorentzian profile with full-width at half maximum (FWHM) equal to $\Gamma^{\mathrm{J}}(\hbar\omega)$. The inhomogeneous broadening due to disorder is described by a Gaussian with standard deviation given by δ^{J}. These two contributions to the broadening are described by a Voigt-profile, $\mathcal{V}$, centered at the energy of the **n**-th COM exciton state. This implies that the optical absorption in Equation (S18) needs to be averaged by weighting with[2]

$$w_X(\hbar\omega, \boldsymbol{n}, \boldsymbol{L}) = \sum_{J=HH,LH} a^J \mathcal{V}\big(\hbar\omega - E^{\mathrm{J}} - E_{\boldsymbol{n}}^{\mathrm{J}}(\boldsymbol{L}); \Gamma^{\mathrm{J}}(\hbar\omega), \delta^{\mathrm{J}}\big). \tag{S22}$$

Analogous to our earlier work, the transient optical absorption spectrum due to excitons is described by combining Equations (S18) - (S22), leading to[2]

$$S_X(\hbar\omega) = \sum_n \int d\boldsymbol{L} D(\boldsymbol{L})\, f_{\boldsymbol{n}}(\boldsymbol{L})\, w_X(\hbar\omega, \boldsymbol{n}, \boldsymbol{L})\big[1 - f_{X,\mathrm{EM}}(t)\, e^{-\beta\hbar\omega}\big]\, n_{\mathrm{NPL}} d. \tag{S23}$$

Comparison of Equations (S23) and (S18) shows that $\sigma_X(\hbar\omega, \bar{N}_X = 0) = \sum_n \int d\boldsymbol{L} D(\boldsymbol{L})\, f_{\boldsymbol{n}}(\boldsymbol{L})\, w_X(\hbar\omega, \boldsymbol{n}, \boldsymbol{L})$.[2] Note, that contributions of internally excited excitons can be neglected, since the oscillator strength is much smaller than for ground state excitons and their population is negligible.[2, 6] The values of the parameters a^J, $\Gamma^{\mathrm{J}}(\hbar\omega)$ and δ^{J} were taken from our previous fits to steady-state abosprton and photoluminescence spectra of the same NPLs as studied here.[2]

S5. Transient absorption due to charge carriers

We now consider the transient absorption due to holes in the heavy hole valence band (VB) and electrons in the conduction band (CB) with wave vector k, and angular momentum projection $m_{j_h} = \pm 3/2$ for holes and $m_{j_e} = \pm 1/2$ for electrons. Transitions from the light hole VB are not taken into account, since these occur at energies above 2.8 eV [see Figure 1(a) of the main text], which is larger than the maximum probe photon energy used in the transient absorption experiments, see Figure 1(b) of the main paper. Below we denote the probability averaged over all NPLs that an electron occupies a state in the VB as $f_v(k, m_{j_h})$ and for an electron in the CB as $f_c(k, m_{j_e})$. The net rate of photon absorption and emission in the presence of charge carriers, analogous to Equation (S10) for excitons, is

$$\langle \Gamma_{\gamma \leftrightarrow e,h} \rangle = \langle \Gamma_{\gamma \rightarrow e,h} \rangle - \langle \Gamma_{e,h \rightarrow \gamma} \rangle$$

$$= \Gamma_{cont}(\hbar\omega) \sum_k{}' \sum_{m_{j_e}, m_{j_h}} \sum_{N_\gamma = 0}^{\infty} \delta_{|m_{j_h} - m_{j_e}|,1} \left[N_\gamma f_v(k, m_{j_h})\left(1 - f_c(k, m_{j_e})\right) - f_c(k, m_{j_e})\left(1 - f_v(k, m_{j_h})\right)(N_\gamma + 1)\right] P_{N_\gamma}(\hbar\omega). \tag{S24}$$

After optical excitation by the laser pump pulse, the factor $N_\gamma f_v(k)\big(1 - f_c(k)\big)$ describes reduced optical absorption due to removal of an electron from the VB and/or population of an electron state in the CB (Pauli blocking), while $f_c(k)\big(1 - f_v(k)\big)\big(N_\gamma + 1\big)$ is due to emission of a photon via recombination of an electron and a hole. The prime ($'$) in the summation over k indicates that only electrons with a wave vector such that the energy difference between the CB and VB

states equals the probe photon energy $\hbar\omega$ contribute to the Equation (S24). Note that in Equation (S24) $\Gamma_{cont}(\hbar\omega)$ takes into account the optical oscillator strength. By defining the hole probability $f_h(k) = 1 - f_v(-k)$ we can rewrite Equation (S24) as

$$\langle \Gamma_{\gamma\leftrightarrow e,h}\rangle = \Gamma_{cont}(\hbar\omega)\sum_k{}' \sum_{m_{je},m_{jh}} \sum_{N_\gamma=0}^{\infty} \delta_{|m_{jh}-m_{je}|,1} \left[N_\gamma \left(1 - f_h(-k,m_{jh}) - f_c(k,m_{je})\right) - f_c(k,m_{je})f_h(-k,m_{jh})\right] P_{N_\gamma}(\hbar\omega). \tag{S25}$$

To connect with the notation in Equations (S12-S15) we recall that the average number of probe photons interacting with a NPL [see Equation (S11)] is

$$\bar{N}_\gamma(\hbar\omega) = \sum_{N_\gamma=0}^{\infty} N_\gamma P_{N_\gamma}(\hbar\omega), \tag{S26}$$

while the number of allowed optical transitions in a NPL at probe photon energy $\hbar\omega$ is

$$N_{trans}(\hbar\omega) = \sum_k{}' \sum_{m_{je},m_{jh}} \delta_{|m_{jh}-m_{je}|,1}, \tag{S27}$$

and the average number of electrons and holes Pauli blocking the optical transitions in a NPL are

$$\bar{N}_e(\hbar\omega, x) = \sum_k{}' \sum_{m_{je},m_{jh}} \delta_{|m_{jh}-m_{je}|,1}\, f_c(k,m_{je}) \tag{S28}$$

$$\bar{N}_h(\hbar\omega, x) = \sum_k{}' \sum_{m_{je},m_{jh}} \delta_{|m_{jh}-m_{je}|,1}\, f_h(-k,m_{jh}), \tag{S29}$$

and finally the average number of photons emitted from a NPL due to the recombination of an electron-hole pair is

$$\bar{N}_{e,h}(\hbar\omega, x) = \sum_k{}' \sum_{m_{je},m_{jh}} \delta_{|m_{jh}-m_{je}|,1}\, f_c(k,m_{je}) f_h(-k,m_{jh}). \tag{S30}$$

Note that the quantities above depend on the position x due to the reduction of the absorbed pump photons (and thus of excitons, electrons and holes) along the path in the sample. By using the definitions above, we now get [analogous to Equation (S13)]

$$\frac{1}{I(\hbar\omega,x)}\frac{dI(\hbar\omega,x)}{dx} = -\frac{1}{\overline{N}_\gamma(\hbar\omega,x)}\frac{\langle \Gamma_{\gamma\leftrightarrow e,h} \rangle}{c} V\, n_{\mathrm{NPL}} = \\ = -\sigma_{cont}(\hbar\omega,\overline{N}_e,\overline{N}_h)\, n_{\mathrm{NPL}}\left[\left(1-\frac{\overline{N}_e(\hbar\omega,x)+\overline{N}_h(\hbar\omega,x)}{N_{trans}(\hbar\omega)}\right) - \frac{\overline{N}_{e,h}(\hbar\omega,x)}{\overline{N}_\gamma(\hbar\omega,x)N_{trans}(\hbar\omega)}\right], \tag{S31}$$

where $\sigma_{cont}(\hbar\omega,\overline{N}_e,\overline{N}_h) = N_{trans}(\hbar\omega)\Gamma_{cont}(\hbar\omega)V/c$ is the absorption cross section of the probe photons. By integrating Equation (S31) over the optical path length, d, we obtain

$$\ln\left(\frac{I(\hbar\omega,d)}{I(\hbar\omega,0)}\right) = -\sigma_{cont}(\hbar\omega,\overline{N}_e,\overline{N}_h)\, n_{\mathrm{NPL}} d\left[1-\frac{1}{d}\int_0^d \left[\frac{\overline{N}_e(\hbar\omega,x)+\overline{N}_h(\hbar\omega,x)}{N_{trans}(\hbar\omega)}\right]dx - \frac{1}{d}\int_0^d \left[\frac{\overline{N}_{e,h}(\hbar\omega,x)}{\overline{N}_\gamma(\hbar\omega,x)N_{trans}(\hbar\omega)}\right]dx\right]. \tag{S32}$$

Hence, the second term in Equation (S6), describing the effect of free electrons and holes on the transient optical absorption after the pump pulse, is found to be

$$S_{cont}(\hbar\omega) = \sigma_{cont}(\hbar\omega,\overline{N}_e,\overline{N}_h)\, n_{\mathrm{NPL}}\, d\left[1-\frac{1}{d}\int_0^d \left[\frac{\overline{N}_e(\hbar\omega,x)+\overline{N}_h(\hbar\omega,x)}{N_{trans}(\hbar\omega)}\right]dx - \frac{1}{d}\int_0^d \left[\frac{\overline{N}_{e,h}(\hbar\omega,x)}{\overline{N}_\gamma(\hbar\omega,x)N_{trans}(\hbar\omega)}\right]dx\right]. \tag{S33}$$

Analogous to our treatment of excitons, we assume a Maxwell-Boltzmann distribution of the charge carriers over energy and take into account their decay during time by a factor $f_q(t,x)$, so that we can write

$$\overline{N}_e(\hbar\omega,x) = f_q(t,x)\, e^{-\beta\left(\frac{m_h}{M}\hbar\omega-\mu_e\right)} \\ \overline{N}_h(\hbar\omega,x) = f_q(t,x)\, e^{-\beta\left(\frac{m_e}{M}\hbar\omega-\mu_h\right)}, \tag{S34}$$

with μ_e and μ_h the chemical potential of electrons and holes, respectively. Scattering of electrons and holes between different k values occurs on a timescale comparable to or less than the oscillation period of optical phonons (~150 fs). This timescale is within the time resolution of our experiments, which is roughly the sum of the pump and probe pulse durations (~400 fs). Therefore, we assume that the initial correlation of the k vectors of an electron and a hole produced by absorption of a photon is lost and we can make the approximation

$$\overline{N}_{e,h}(\hbar\omega,x) = \overline{N}_e(\hbar\omega,x)\,\overline{N}_h(\hbar\omega,x). \tag{S35}$$

By combining Equations (S34) and (S35) we get

$$S_{cont}(\hbar\omega) = \sigma_{cont}(\hbar\omega, \bar{N}_e, \bar{N}_h)\, n_{\mathrm{NPL}} d \left[1 - \left[e^{-\beta\left(\frac{m_h}{M}\hbar\omega - \mu_e\right)} + e^{-\beta\left(\frac{m_e}{M}\hbar\omega - \mu_h\right)} \right] \frac{1}{d} \int_0^d \frac{f_q(t,x)}{N_{trans}(\hbar\omega)} dx \right.$$

$$\left. - e^{-\beta(\hbar\omega - \mu_e - \mu_h)} \frac{1}{d} \int_0^d \frac{f_q^2(t,x)}{\bar{N}_\gamma(\hbar\omega, x) N_{trans}(\hbar\omega)} dx \right]. \quad \text{(S36)}$$

For equal densities of electrons and holes, their chemical potentials are related by[7]

$$\mu_h(\mu_e) = \beta^{-1} \ln\left[\left(1 + e^{\beta\mu_e}\right)^{m_e/m_h} - 1 \right]. \quad \text{(S37)}$$

Note that we ignore the discreteness of electron and hole states due to the finite lateral deimensions of the NPLs. This is a good approximation, since according to recent theoretical modeling the energetic spacing between the electronic states in CdSE NPLS is a few meV,[8] which is comparible to energetic broading resulting from dephasing by e.g. electron-phonon scattering.[9] In the classical regime the term $\beta\mu_e$ is very negative, so that (S37) leads to

$$\Delta\mu = \mu_h - \mu_e \simeq \ln\left(\frac{m_e}{m_h}\right) \beta^{-1}. \quad \text{(S38)}$$

By defining factors that take into account the decay of charge carriers during time as

$$f_{C_1}(t) = \frac{e^{\beta\mu_e}}{N_{trans}(\hbar\omega)\, d} \int_0^d f_q(t,x)\, dx$$

$$f_{C_2}(t) = \frac{e^{\beta(\mu_e + \mu_h)}}{N_{trans}(\hbar\omega)\, d} \int_0^d \frac{f_q^2(t,x)}{\bar{N}_\gamma(\hbar\omega, x)} dx. \quad \text{(S39)}$$

we get from Equation (S36)

$$S_{cont}(\hbar\omega) = \sigma_{cont}(\hbar\omega, \bar{N}_e, \bar{N}_h)\, n_{\mathrm{NPL}} d \left[1 - f_{C_1}(t) \left[e^{-\beta\left(\frac{m_h}{M}\hbar\omega\right)} + e^{-\beta\left(\frac{m_e}{M}\hbar\omega - \Delta\mu\right)} \right] - f_{C_2}(t) e^{-\beta\hbar\omega} \right], \quad \text{(S40)}$$

where $\Delta\mu$ is calculated according to the approximation in Equation (S38). Comparing Equation (S40) with (S6) yields

$$f_{cont}(\hbar\omega, \bar{N}_e, \bar{N}_h) = f_{C_1}(t) \left[e^{-\beta\left(\frac{m_h}{M}\hbar\omega\right)} + e^{-\beta\left(\frac{m_e}{M}\hbar\omega - \Delta\mu\right)} \right] + f_{C_2}(t) e^{-\beta\hbar\omega}. \quad \text{(S41)}$$

In Equations (S40) and (S41) the term with the factor $f_{C_1}(t)$ results from effects of Pauli blocking by charge carriers in the valence and conduction bands, as well as electron-hole recombination by stimulated emission. The factor $f_{C_2}(t)$ is due to spontaneous radiative electron-hole recombination.

For fitting to the experimental data, Equation (S40) must be averaged over the NPL size distribution and broadened by inclusion of a Voigt profile (analogous to Equation (S22) for excitons in Section S4). For the combined electron and hole continua we get

$$w_{cont}(\hbar\omega, \boldsymbol{n}, \boldsymbol{L}) = \int_{-\infty}^{+\infty} d\epsilon_0 \mathcal{V}(\hbar\omega - \epsilon_0; \Gamma_C^{\mathrm{HH}}(\hbar\omega), \delta_C^{\mathrm{HH}})\ \Theta[\epsilon_0 - E_X^{HH} - E^{HH} - E_{\boldsymbol{n}}^{HH}(\boldsymbol{L})], \tag{S42}$$

where $\Gamma_C^{\mathrm{HH}}(\hbar\omega)$ and δ_C^{HH} represent the homogeneous and inhomogeneous broadening of transitions to the continuum, respectively, $\Theta(x)$ is the Heaviside step-function and E_X^{HH} is the HH exciton binding energy (here defined positive). Averaging Equation (S34) using the relative weights of Equation (S35) yields

$$S_{cont}(\hbar\omega) = b_{cont} \sum_{\boldsymbol{n}} \int d\boldsymbol{L}\, D(\boldsymbol{L})\, w_{cont}(\hbar\omega, \boldsymbol{n}, \boldsymbol{L})[1 - f_{cont}(\hbar\omega, \bar{N}_e, \bar{N}_h)], \tag{S43}$$

where b_{cont} is a constant analogous to a^J in Equation (S23).

S6. Transient absorption features PA_1 and PA_2 due to biexcitons and trions

The absorbance features (PA_1 and PA_2) in the ΔA spectra in Figures 2 and 4 of the main text could be described by Gaussians centered energy E_{G_i} with standard deviation δ_{G_i} (i=1,2). We attribute these features to formation of biexcitons and trions by absorption of a probe photon near a HH exciton or a charge carrier produced by the pump pulse, as further discussed below and in the main text. We found from our fits that E_{G_i} and δ_{G_i} depend on time. We attribute this to the time-dependence of the number of excitons and charge carriers.

S7. Total transient absorbance

Taking into account the effects of excitons, charge carriers and biexcitons/trions we find that the total transient absorption signal is given by

$$S(\hbar\omega) = S_X(\hbar\omega) + S_{cont}(\hbar\omega) + S_{PA_1}(\hbar\omega) + S_{PA_2}(\hbar\omega). \tag{S44}$$

In what follows we define the probe photon energy by $E_p = \hbar\omega$. Combining Equations (S5), (S23), (S36) and adding the contribution of biexcitons/trions (Section S6) the absorbance after the pump laser pulse is found to be

$$\begin{aligned} A_{on}(E_p, t) = A_X(E_p) + C_{off}(E_p) - A_X\left(E_p + \Delta E(t)\right) e^{-\beta[E_p + \Delta E(t)]} f_{X,\mathrm{EM}}(t) \\ - C_1\left(E_p + \Delta E(t)\right) f_{C_1}(t) - C_2\left(E_p + \Delta E(t)\right) f_{C_2}(t) \\ + G_1\left(E_p, E_{G_1}(t), \delta_{G_1}(t)\right) f_{G_1}(t) + G_2\left(E_p, E_{G_2}(t), \delta_{G_2}(t)\right) f_{G_2}(t). \end{aligned} \tag{S45}$$

Note that a time-dependent energy shift, $\Delta E(t)$, was needed to describe the presence of a double absorbance feature in A_{on} at early times after HH excitation, and a high-energy shoulder of the HH peak after LH and continuum excitation (see Figure S3). We attribute this shift due to Coulomb screening by free charge carriers or filling of charge traps (via free charges or exciton quenching). In Equation (S45), the first two terms represent the ground state absorbance, which is equal to $A_0(E_p) = A_X(E_p) + C_{off}(E_p)$. The third term containing the factor $-A_X\left(E_p + \Delta E(t)\right)$ describes bleach due to (spontaneous) photon emission from excitons. The fourth and fifth terms with factors $-C_1\left(E_p + \Delta E(t)\right)$ and $-C_2\left(E_p + \Delta E(t)\right)$ take into account bleach due to Pauli blocking by holes in the valence band and electrons in the conduction band, as well as their radiative recombination. We attribute the sixth term with the Gaussian function $G_1\left(E_p, E_{G_1}(t), \delta_{G_1}(t)\right)$ to the probe pulse producing a HH exciton near a HH exciton or a charge carrier produced by the pump pulse. The probe pulse then forms a HH-HH biexciton or a trion consisting of a HH exciton and an electron or a hole. The last term with the Gaussian function $G_2\left(E_p, E_{G_2}(t), \delta_{G_2}(t)\right)$ is attributed to contributions from the high-energy absorbance tail due to formation of a HH-HH biexciton by the probe pulse, or formation of a LH-HH biexciton.

Comparing Equation (S45) with Equations (S23), (S41) and (S43) it can be seen that

$$A_X(E_p) = \tilde{a}_X \sum_n \int d\boldsymbol{L}\, D(\boldsymbol{L}) f_{\boldsymbol{n}}(\boldsymbol{L})\; w_X(E_p, \boldsymbol{n}, \boldsymbol{L}), \tag{S46}$$

$$C_{off}(E_p) = \tilde{b}_{cont} \sum_n \int d\boldsymbol{L}\, D(\boldsymbol{L})\; w_{cont}(E_p, \boldsymbol{n}, \boldsymbol{L}) \tag{S47}$$

$$A_X\left(E_p + \Delta E(t)\right) = \tilde{a}_X \sum_n \int d\boldsymbol{L}\, D(\boldsymbol{L}) f_{\boldsymbol{n}}(\boldsymbol{L})\; w_X(E_p + \Delta E(t), \boldsymbol{n}, \boldsymbol{L}), \tag{S48}$$

$$C_1\left(E_p + \Delta E(t)\right) = \\ = \tilde{b}_{cont} \sum_n \int d\boldsymbol{L}\, D(\boldsymbol{L})\; w_{cont}(E_p + \Delta E(t), \boldsymbol{n}, \boldsymbol{L}) \Big[e^{-\beta\left(\frac{m_h}{M}\left(E_p + \Delta E(t)\right)\right)} \\ + e^{-\beta\left(\frac{m_e}{M}\left(E_p + \Delta E(t)\right) - \Delta\mu\right)} \Big], \tag{S49}$$

$$C_2\left(E_p + \Delta E(t)\right) = \tilde{b}_{cont} \sum_n \int d\boldsymbol{L}\, D(\boldsymbol{L})\; w_{cont}(E_p + \Delta E(t), \boldsymbol{n}, \boldsymbol{L})\; e^{-\beta\left(\left(E_p + \Delta E(t)\right)\right)}, \tag{S50}$$

where $\tilde{a}_X = a_X / \ln(10)$ and $\tilde{b}_{cont} = b_{cont} / \ln(10)$, see Equation (S5).

In conclusion, transient absorbance spectra are modeled using Equation (S45). Analogous to our previous work,[2] the spectral shapes of A_X, C, C_1 and C_2 take into account the COM motion of excitons, the lateral size distribution of NPLs and

(in)homogenous broadening via $w_X(\hbar\omega, \boldsymbol{n}, \boldsymbol{L})$ and $w_{cont}(\hbar\omega, \boldsymbol{n}, \boldsymbol{L})$ in Equations and (S22) and (S43), respectively. The values of the broadening parameters $\Gamma^{\mathrm{J}}(\hbar\omega)$, δ^{J} and $\Gamma_C^{\mathrm{J}}(\hbar\omega)$ and δ_C^{J} are taken from our previous study.[2] Note that the spectral shape of $w_i(E_p, \boldsymbol{n}, \boldsymbol{L})$ is identical to that of $w_i(E_p + \Delta E(t), \boldsymbol{n}, \boldsymbol{L})$ with the latter only being shifted by $\Delta E(t)$. Finally, in the fit of Equation (S45 to the experimental data the factors in Equations (S48)-(S50) and the Gaussian functions in Equation (S45) the factors in Equation (S45) are normalized to their maximum and then multiplied by the functions $f_i(t)$, where i = EM, C_1, C_2, G_1, G_2. The functions $f_i(t)$ take into account the decay of excitons and charge carriers by photon emission and non-radiative processes (Auger recombination, trapping) in a phenomenological way.

$\Delta E(t)$	Energy shift due to Coulomb screening and filling of charge traps
$f_{X,\mathrm{EM}}(t)$	Decay of excitons
$f_{C_1}(t), f_{C_2}(t)$	Decay of charge carriers
$f_{G_1}(t), f_{G_2}(t)$	Decay of TA due to biexcitons/trions
$E_{G_1}(t)$, $E_{G_2}(t)$	Peak energies of the biexciton/trion features
$\delta_{G_1}(t)$, $\delta_{G_2}(t)$	Spectral width of the biexciton/trion features

Table S1. Fitting parameters in Equation (S45). The time-dependence of the parameters obtained from fitting to experimental data is shown in Figure S4.

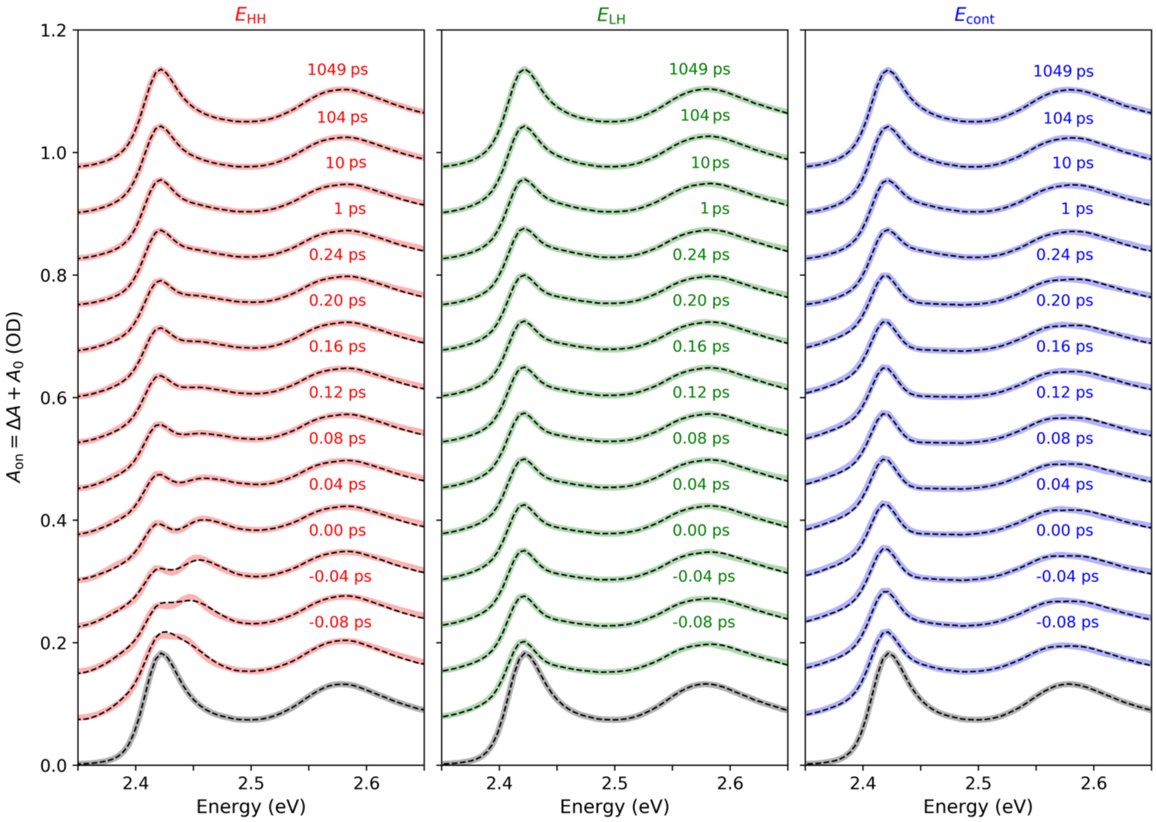

Figure S3. Time evolution of A_{on} spectra after excitation at E_{HH} (left column, red), E_{LH} (middle column, blue) and E_{cont} (right column, green). Spectra were obtained for similar N_{av}~3 [squares in Figure S1]. After E_{HH} excitation, we resolve a double peak at early time. This, as well as the portion of the spectrum between the HH and LH peak at higher excitation energies, can be modeled by taking into account a time-dependent energy shift ΔE due to Coulomb screening and trap filling [see Equation (S45) in Section S6]. The results obtained from fits of Equation (S45) are shown as dashed black curves. The grey curve at the bottom of each column is the ground state absorbance spectrum (A_0).

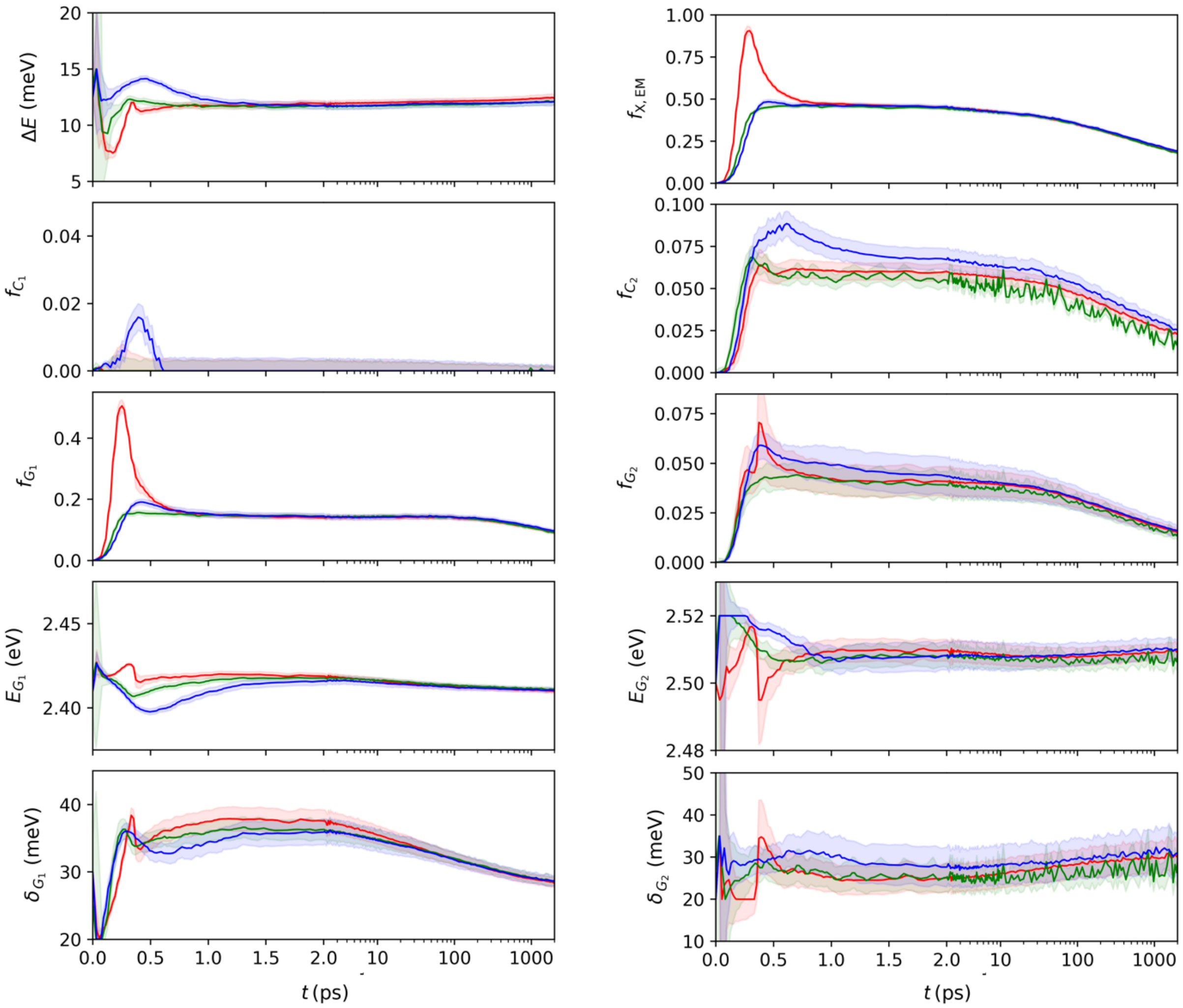

Figure S4. Time-dependence of the fitting parameters (solid lines) in Equation (S45) with the uncertainty represented by the shaded curves.

REFERENCES

(1) Tomar, R.; Kulkarni, A.; Chen, K.; Singh, S.; van Thourhout, D.; Hodgkiss, J. M.; Siebbeles, L. D. A.; Hens, Z.; Geiregat, P. Charge Carrier Cooling Bottleneck Opens up Nonexcitonic Gain Mechanisms in Colloidal CdSe Quantum Wells. *J. Phys. Chem. C* **2019**, *123*, 9640-9650.

(2) Failla, M.; Flórez, F. G.; Salzmann, B. B. V.; Vanmaekelbergh, D.; Stoof, H. T. C.; Siebbeles, L. D. A. Observation of the Quantized Motion of Excitons in CdSe Nanoplatelets. *Phys. Rev. B* **2020**, *102*, 195405.

(3) Fox, M., *Optical Properties of Solids*; Oxford University Press, 2010.

(4) Laikhtman, B. Are Excitons Really Bosons? *J. Phys. Condens. Matter* **2007**, *19*, 295214.

(5) Moskalenko, S. A.; Snoke, D. W., *Bose-Einstein Condensation of Excitons and Biexcitons*; Cambridge University Press, 2005.

(6) García Flórez, F.; Kulkarni, A.; Siebbeles, L. D. A.; Stoof, H. T. C. Explaining Observed Stability of Excitons in Highly Excited CdSe Nanoplatelets. *Phys. Rev. B* **2019**, *100*, 245302.

(7) García Flórez, F.; Siebbeles, L. D. A.; Stoof, H. T. C. Biexcitons in Highly Excited CdSe Nanoplatelets. *Phys. Rev. B* **2020**, *102*, 115302.

(8) Quick, M. T.; Ayari, S.; Owschimikow, N.; Jaziri, S.; Achtstein, A. W. Quantum Nature of THz Conductivity: Excitons, Charges, and Trions in 2D Semiconductor Nanoplatelets and Implications for THz Imaging and Solar Hydrogen Generation. *ACS Applied Nano Materials* **2022**, *5*, 8306-8313.

(9) Achtstein, A. W.; Owschimikow, N.; Quick, M. T. Population Dependence of THz Charge Carrier Mobility and Non-Drude-Like Behavior in Short Semiconductor Nanowires. *Nanoscale* **2022**, *14*, 19-25.
